# Curcuminoids amplify host innate antiviral immunity via the CRYAB‐RBM26 axis in viral infection

**DOI:** 10.1002/imt2.70111

**Published:** 2026-02-24

**Authors:** Tingting Chen, Xiang Wang, Guangyuan Zhang, Lu Wang, Junhao Wan, Lirong Tan, Xinyue Fan, Siqin Meng, Zhixing Jin, Yuzhang Liang, Haoze Li, Ziyuan Wan, Xiaotong Xu, Yan Zhang, Li Qin, Anna Malashicheva, Yu Du, Guangchao Zang, Guixue Wang

**Affiliations:** ^1^ Department of Orthopedics the Second Affiliated Hospital of Chongqing Medical University Chongqing China; ^2^ Institute of Panvascular Biology, JinFeng Laboratory Chongqing China; ^3^ Pathogen Biology and Immunology Laboratory, Lab Teaching & Management Center Chongqing Medical University Chongqing China; ^4^ National Key Laboratory for Development and Utilization of Forest Food Resources, Institute of Chemical Industry of Forest Products, Chinese Academy of Forestry Nanjing China; ^5^ Institute of Medical College, Guizhou University Guiyang China; ^6^ Institute of Cytology, Russian Academy of Science St. Petersburg Russia; ^7^ Key Laboratory of Biorheological and Technology of Ministry of Education, National Local Joint Engineering Laboratory for Vascular Implants Bioengineering College of Chongqing University Chongqing China; ^8^ School of Chinese Medicine, Hubei University of Chinese Medicine Wuhan China

**Keywords:** curcuminoids, innate immune response, mitochondrial DNA, nucleotide metabolism, type I interferon, viral infection

## Abstract

Curcuminoids, including curcumin (CUR) and demethoxycurcumin (DMC), are known for their antiviral properties, but their underlying antiviral targets remain unclear, and the relationship between curcuminoids and the type I interferon (IFN‐I) signaling pathway has not been fully elucidated. Here, we explored the regulatory effects of DMC and CUR on the IFN‐I pathway in an EV‐D68‐infected murine model and employed multiomics analysis to identify key drug targets and their interaction networks. FTIR analysis indicated that DMC has better physicochemical stability than CUR, exhibiting greater stability under changes in light, temperature, and pH. In both in *vitro* and neonatal mouse models, DMC and CUR effectively inhibited EV‐D68 replication by suppressing viral *2A* gene expression and the release of proinflammatory cytokines. Both compounds upregulated the molecular chaperone CRYAB (αB‐crystallin), which translocates to the nucleus and acts as a central regulator of host metabolism and antiviral immunity during EV‐D68 infection. Further multiomics analyses revealed that CRYAB overexpression inhibited purine metabolism and upregulated interferon‐stimulated genes. Proteomic profiling identified RBM26 as a key CRYAB‐interacting target. CRYAB stabilizes RBM26 by inhibiting virus‐induced ubiquitination, which leads to enhanced IFN‐I responses. DMC and CUR activated the mtDNA‐cGAS‐STING pathway via RBM26, stimulating downstream signaling and antiviral effects. RBM26 reconstitution altered the splicing of cytidine/uridine monophosphate kinase 2 (*CMPK2*), resulting in increased nucleotide turnover and reduced cytidine levels, impairing viral replication. DMC/CUR treatment or *CRYAB* overexpression similarly reduced intracellular cytidine and uridine levels, increasing antiviral activity. Additionally, DMC/CUR restored mtDNA levels suppressed by EV‐D68 infection in an RBM26‐dependent manner, stimulating cGAS‐mediated cGAMP production and activating the STING‐TBK1‐IRF3 axis. These findings not only clarify the molecular mechanisms underlying the antiviral effects of curcuminoids but also highlight their therapeutic potential as host‐directed antiviral agents.

## Highlights

- CRYAB‐RBM26 is identified as a core antiviral regulatory axis of curcuminoids.
- Curcuminoids modulate type I interferon signaling in a dual‐phase manner.
- Curcuminoids prevent virus‐induced hijacking of host nucleotide metabolism in an RBM26‐dependent manner.
- Multiomics reveals a compound‐target‐metabolism‐immune cascade that mediates antiviral effects.

## Graphical abstract

DMC/CUR antagonize viral hijacking of host nucleic acid resources and finely regulate the induction and effector phases of the type I interferon (IFN‐I) pathway through the CRYAB‐RBM26 axis. This molecular axis serves as a key downstream regulator for DMC/CUR activation of the IFN‐I pathway.

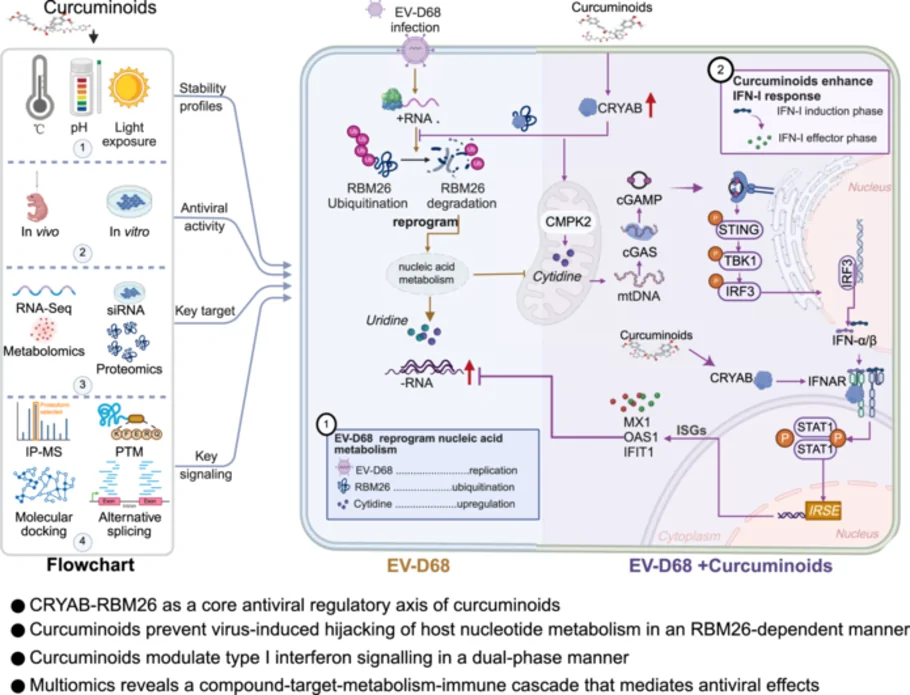

## INTRODUCTION

Turmeric, a traditional spice and coloring agent in South Asian and Middle Eastern cuisines [1], contains curcuminoids such as curcumin (CUR), demethoxycurcumin (DMC), and bisdemethoxycurcumin [2, 3]. Curcumin has broad therapeutic potential and multiple bioactivities, including anti‐inflammatory, antioxidant, antitumour, antibacterial, antiviral, and neuroprotective effects [4]. Previously, we employed hyaluronic acid‐modified curcumin‐based nanomedicines to promote diabetic wound healing [5]. CUR has been reported to inhibit diverse viral infections by suppressing replication or disrupting virus–host interactions [6, 7], but its poor water solubility and pH instability cause rapid degradation into inactive products [8, 9]. DMC also has broad pharmacological potential, including antihypertensive, antimalarial, antiviral [10], and antibacterial effects [11, 12, 13, 14]. Since it lacks a methoxy group on the aromatic ring, DMC is more water soluble at room temperature [15, 16] and shows greater stability and bioactivity than CUR under physiological conditions [17, 18]. However, DMC retains the CUR backbone and exhibits antiviral activity against various viruses, including the inhibition of conformation changes in viral envelope glycoproteins that facilitate attachment to the host cell membrane [19].

Previous studies have shown that DMC significantly enhances the type I interferon (IFN‐I) response and upregulates the expression of interferon‐stimulated geISGnes (ISGs) [20]. Similarly, another study demonstrated that CUR could potentiate type I IFN responses [21]. However, it remains unclear whether this represents a common antiviral mechanism shared by both CUR and DMC. Elucidating these underlying mechanisms is essential for advancing the clinical development of curcuminoid‐based therapeutics and broadening their potential applications in biomedical research. *Enterovirus* D68 (EV‐D68), a member of the *Picornaviridae* family, is a nonenveloped, positive‐sense single‐stranded RNA virus [22, 23, 24, 25]. EV‐D68 mainly causes respiratory illnesses, particularly in immunocompromised individuals such as children and elderly individuals. Moreover, infection can result in neurological complications, including acute flaccid myelitis [26, 27, 28, 29], whose early symptoms often include headache, stiffness, or pain in the neck and back. In more severe cases, the condition may progress to neurological dysfunction and muscle weakness [30, 31]. Currently, there are no approved antiviral therapies or vaccines available for EV‐D68 infection.

Therefore, this study used comparative analyses to assess their physicochemical properties and distinct cellular responses. Proteomic profiling and molecular docking were utilized to identify central hub targets associated with the antiviral activities of CUR and DMC. A protein library regulated by these targets was established, and metabolomic analyses were further integrated to delineate the common metabolic pathways regulated by both compounds. On the basis of a comprehensive drug–target–metabolic pathway network, this study elucidated the mechanism by which curcuminoids modulate the reprogramming of host metabolism during viral infection. These findings provide novel insights into the interactions between curcuminoids and the host antiviral response, addressing a knowledge gap regarding natural product‐based modulation of host‐targeted pathways against RNA viral infection.

## RESULTS

### Compared with CUR, DMC has better structural stability and antiviral efficacy

To investigate the antiviral mechanism of CUR and DMC, we first characterized the structural features of CUR and DMC via Fourier‐transform infrared spectroscopy (FTIR). The results revealed that the spectrum of DMC lacked several characteristic absorption peaks observed in CUR, specifically at 1150.6, 1105.5, and 1423.4 cm^−1^. This is because CUR contains an additional –OCH₃ group on its benzene ring compared with DMC, which gives rise to the characteristic absorption peaks observed in CUR, specifically at 1150.6 cm^−1^ (C–O stretching vibration of –OCH_3_ in CUR, typical range: 1300–1000 cm^−1^), 1105.5 cm^−1^ (C–O stretching vibration of the benzene ring in CUR, typical range: 1150‐1060 cm^−1^), and 1423.4 cm^−1^ (C–H bending vibration of –OCH_3_ in CUR, typical range: 1465–1340 cm^−1^) (Figure 1A). Next, the stability of both compounds was assessed under various environmental conditions, including different temperatures, light exposure durations, and pH levels. Compared with CUR, DMC exhibited markedly greater stability, demonstrating better resistance to degradation upon prolonged light exposure (>50 min), as well as under strongly acidic (pH < 3) and alkaline (pH > 8) conditions and at temperatures exceeding 30°C (Figure 1B). These results suggest that DMC is more stable under extreme conditions than that of CUR.

**Figure 1:**
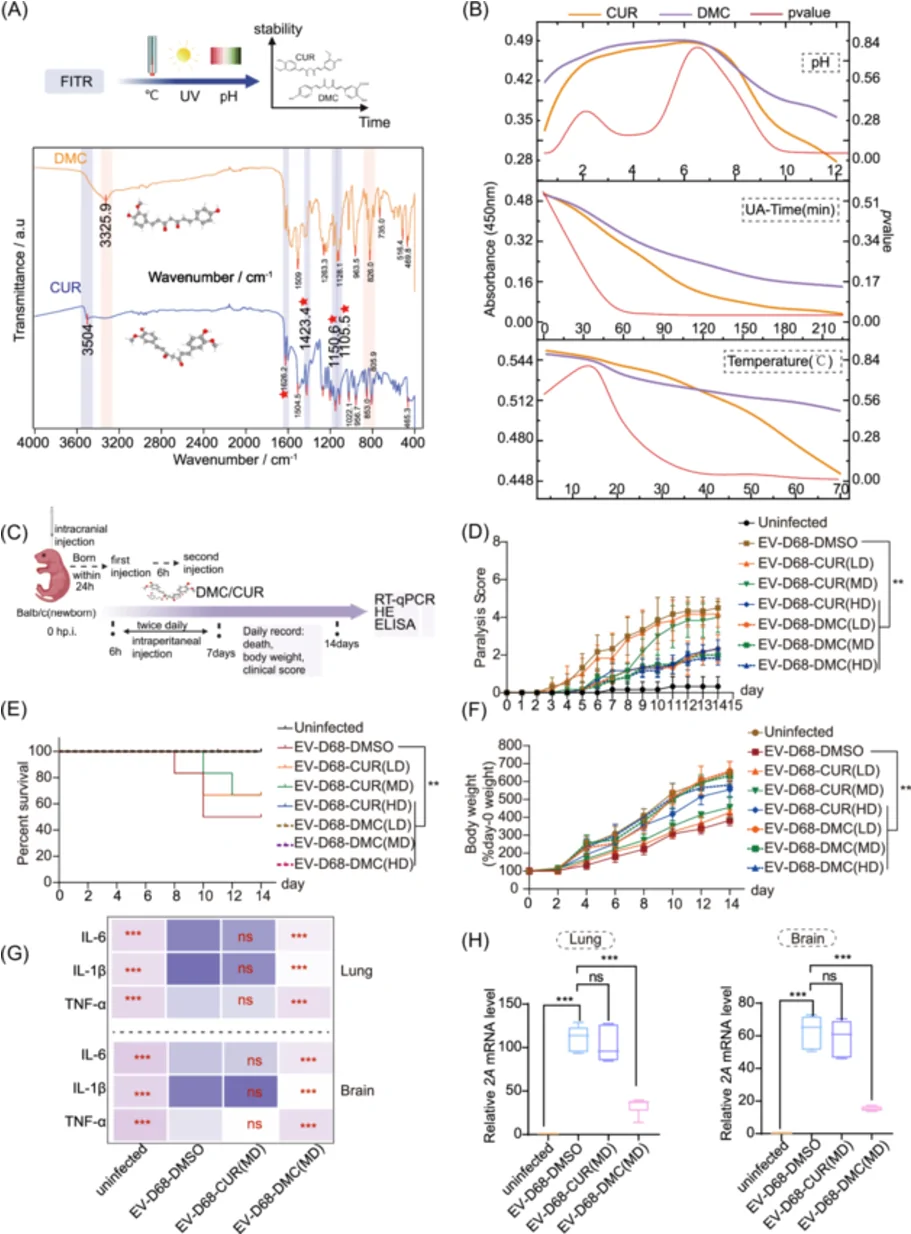
Stability of CUR and DMC and their in vivo antiviral efficacy against EV‐D68. (A) Fourier transform infrared spectroscopy (FTIR) analysis of CUR and DMC. (B) Stability assessment of CUR and DMC by measuring the absorbance at 450 nm over time. (C) Schematic of the neonatal mouse model used to evaluate in vivo antiviral activity. BALB/c mice were intracranially injected with EV‐D68 (1 × 10^7^ PFU) or PBS (mock) within 24 h of birth. At 6 h postinfection (hpi), the mice were treated intraperitoneally with CUR or DMC daily for 7 days. The mice (*n* = 6 per group) were monitored for signs of encephalitis until Day 14. All procedures were approved by the institutional Animal Care and Use Committee. (D) Paralysis scores recorded in EV‐D68‐infected mice following CUR or DMC treatment. The graphs show the mean ± SD, *n* = 6. **p < 0.05; ***p < 0.001. (E) Survival curves of EV‐D68‐infected mice following DMC or CUR treatment. (F) Body weight changes in EV‐D68‐infected mice treated with DMC or CUR were recorded over the observation period. The data represent three independent experiments. The graphs show the mean ± SD, *n* = 6. **p < 0.05; ***p < 0.001. (G) Heatmap of the serum cytokine levels measured via ELISA. The MOCK group included untreated control mice. All the data represent three independent experiments. The results are expressed as the mean ± SD. Statistical significance: **p < 0.05, ***p < 0.001, ns, not significant. (H) Relative mRNA levels of the viral *2A* gene in lung tissues at 14 days post‐infection (dpi), as quantified by RT‐qPCR. The results are expressed as the mean ± SD, *n* = 3. Statistical significance: **p < 0.05, ***p < 0.001, ns, not significant.

To further investigate the antiviral activity of CUR and DMC, we used a neonatal BALB/c mouse model infected with EV‐D68 within 24 h of birth. The mice were subsequently treated with low, medium, or high doses of CUR or DMC (Figure 1C). According to paralysis scores and survival rates, DMC demonstrated effective antiviral activity even at low doses, with dose‐independent protection, whereas CUR showed significant antiviral effects only at high doses (*p* < 0.05) (Figure 1D,E). Additionally, body weight analysis revealed that EV‐D68 infection caused significant growth retardation in infected mice compared with that in the control group (*p* < 0.05). Both the DMC and CUR treatments effectively alleviated the growth inhibition caused by viral infection, although CUR required a higher effective concentration than that of DMC (Figure 1F). Histological analysis of brain, lung, and liver tissues revealed reduced inflammation in the CUR (HD) and DMC (MD) groups, and DMC (MD) had more pronounced effects (Figure S1A). Notably, inflammation was more prominent in the brain and lungs than in the liver and spleen. Therefore, we assessed the levels of proinflammatory cytokines (IL‐1β, IL‐6, and TNF‐α) in brain and lung tissues. At equivalent drug concentrations, only DMC markedly reduced cytokine levels, whereas CUR slightly reduced cytokine levels (Figure 1G).

The EV‐D68 2A protein interacts with the host cell translation machinery to promote viral genome replication [32]. Therefore, we quantified the transcript levels of the viral *2A* gene in lung and brain tissues via RT‐qPCR. Compared with DMSO, DMC markedly reduced *2A* gene expression (*p* < 0.0001), whereas CUR exhibited only a weak and nonsignificant inhibitory effect under the same conditions (Figure 1H and Figure S1B). Overall, these data highlight the better stability and antiviral efficacy of DMC than CUR.

### The antiviral effects of DMC and CUR against EV‐D68 are associated with an enhanced innate immune response and CRYAB expression

The cytotoxicity and antiviral efficacy of these compounds were further evaluated in Rhabdomyosarcoma Cells (RD cells) infected with EV‐D68. DMC showed marginally greater cytotoxicity than CUR (IC_50_ 23.79 ± 4.22 μM vs. 30.20 ± 3.21 μM), yet it inhibited viral *2A* gene expression more potently (IC_50_ 4.51 ± 0.88 μM vs. 15.45 ± 1.25 μM) (Figure 2A).

**Figure 2:**
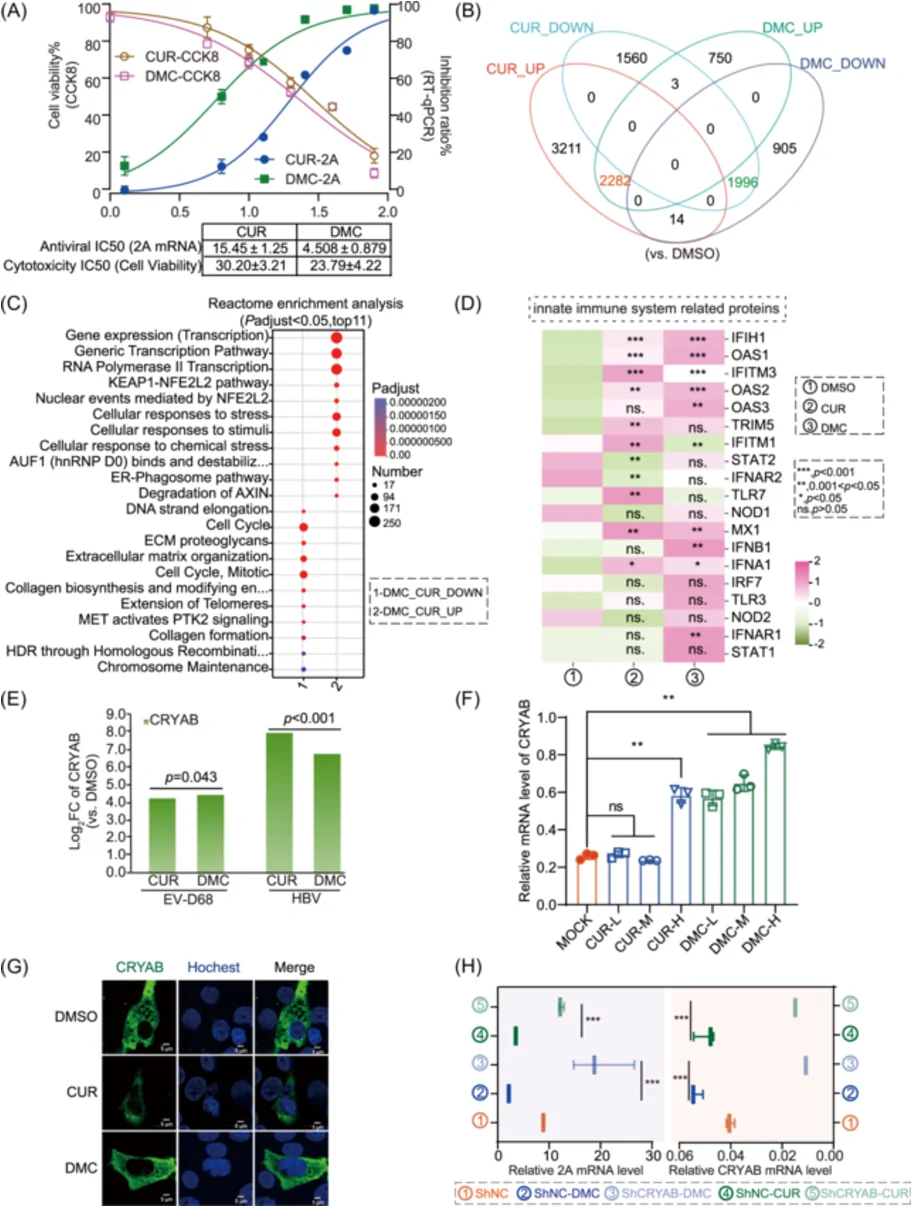
Identification of the molecular targets and antiviral mechanisms of CUR and DMC. (A) Quantification of viral *2A* gene expression in EV‐D68‐infected RD cells treated with CUR or DMC. IC_50_ values were determined for both antiviral efficacy and cytotoxicity. (B) Venn diagram illustrating differentially expressed genes (DEGs) commonly regulated by CUR and DMC on the basis of RNA‐seq data. (C) Reactome pathway enrichment analysis of shared upregulated and downregulated DEGs. Red arrows indicate pathways enriched among upregulated genes; green arrows indicate downregulated gene‐associated pathways. (D) Heatmap of gene expression levels related to innate immune signaling across treatment groups. (E) Transcriptomic analysis of *CRYAB* expression in both RD‐EV‐D68 and HepG2‐HBV‐infected models following treatment. (F) RT‐qPCR analysis of CRYAB mRNA expression in RD cells treated with various concentrations of CUR or DMC for 48 h. DMSO served as the vehicle control (MOCK). The data are presented as the mean ± SD, *n* = 3. Statistical significance: **p < 0.05, ***p < 0.001, ns, not significant. (G) Immunofluorescence analysis of CRYAB protein localization in RD cells after treatment with CUR or DMC. DMSO‐treated cells were used as controls. (H) RT‐qPCR quantification of *2A* gene expression after CRYAB knockdown. RD cells were transfected with shCRYAB or negative control shRNA (shNC), followed by EV‐D68 infection. After 2 h, the media was replaced with CUR‐ or DMC‐containing media. Total RNA was extracted 24 h later for RT‐qPCR. The data are presented as the mean ± SD, *n* = 3. Significant differences are indicated by different letters, whereas no significant differences are indicated by the same letter.

Although the antiviral effective concentrations of CUR and DMC differ, both compounds effectively inhibited the virus. RNA‐seq profiling was performed to identify potential common targets modulated by both drugs during EV‐D68 infection, revealing 2282 upregulated and 1996 downregulated genes shared between the two treatment groups (Figure 2B). Reactome pathway enrichment analysis revealed that the upregulated genes were associated with gene expression, cellular stress responses, interferon signaling, and ER–phagosome pathways, whereas the downregulated genes were predominantly associated with DNA strand elongation processes (Figure 2C). Heatmap profiling of innate immune genes revealed that *IFIH1*, *OAS1*, *IFITM3*, *IFNA1*, and *OAS2* were upregulated in both the CUR and DMC treatment groups (Figure 2D). Among the shared upregulated genes, CRYAB was consistently increased in both the RD‐EV‐D68 and HepG2‐HBV models after DMC treatment (Figure 2E). RT‐qPCR confirmed that DMC evoked a significantly greater upregulation of CRYAB mRNA than CUR did in RD cells (Figure 2F). Cycloheximide (CHX) chase experiments demonstrated that, compared with the positive control MG132, DMC/CUR did not significantly affect the stability of the CRYAB protein (Figure S2A). Additionally, curcumin has been shown to regulate HSF1 activity, inducing its nuclear translocation and binding to DNA to activate the expression of HSPs [33, 34, 35]. Furthermore, compared with DMC/CUR treatment alone, the addition of an HSF1 inhibitor significantly downregulated CRYAB transcription, indicating that the upregulation of *CRYAB* by DMC/CUR is HSF1‐dependent (Figure S2B). Moreover, the molecular docking results revealed that both DMC and CUR strongly bind to the HSF1 protein (Figure S2C). Similar transcriptomic responses were observed in the HepG2‐HBV infection model (Figure S3A). Molecular docking predicted binding affinities of −6.127 kcal/mol for CUR and −6.348 kcal/mol for DMC toward CRYAB, with DMC interacting with more residues (SER53, PRO16, ARG12, ALA57, TRP60, ARG11) than that of CUR (ARG12, ALA57, TRP60, ARG11) (Figure S3B). Nevertheless, immunofluorescence (IF) analysis indicated that neither compound altered the subcellular localization of CRYAB (Figure 2G). Compared with sh‐NC, shRNA‐mediated CRYAB depletion in CUR‐ or DMC‐treated RD cells led to increased viral *2A* gene expression, suggesting that CRYAB is indeed functionally involved in the antiviral activity of both curcuminoids (Figure 2H, Figure S2D,E).

**Figure 3:**
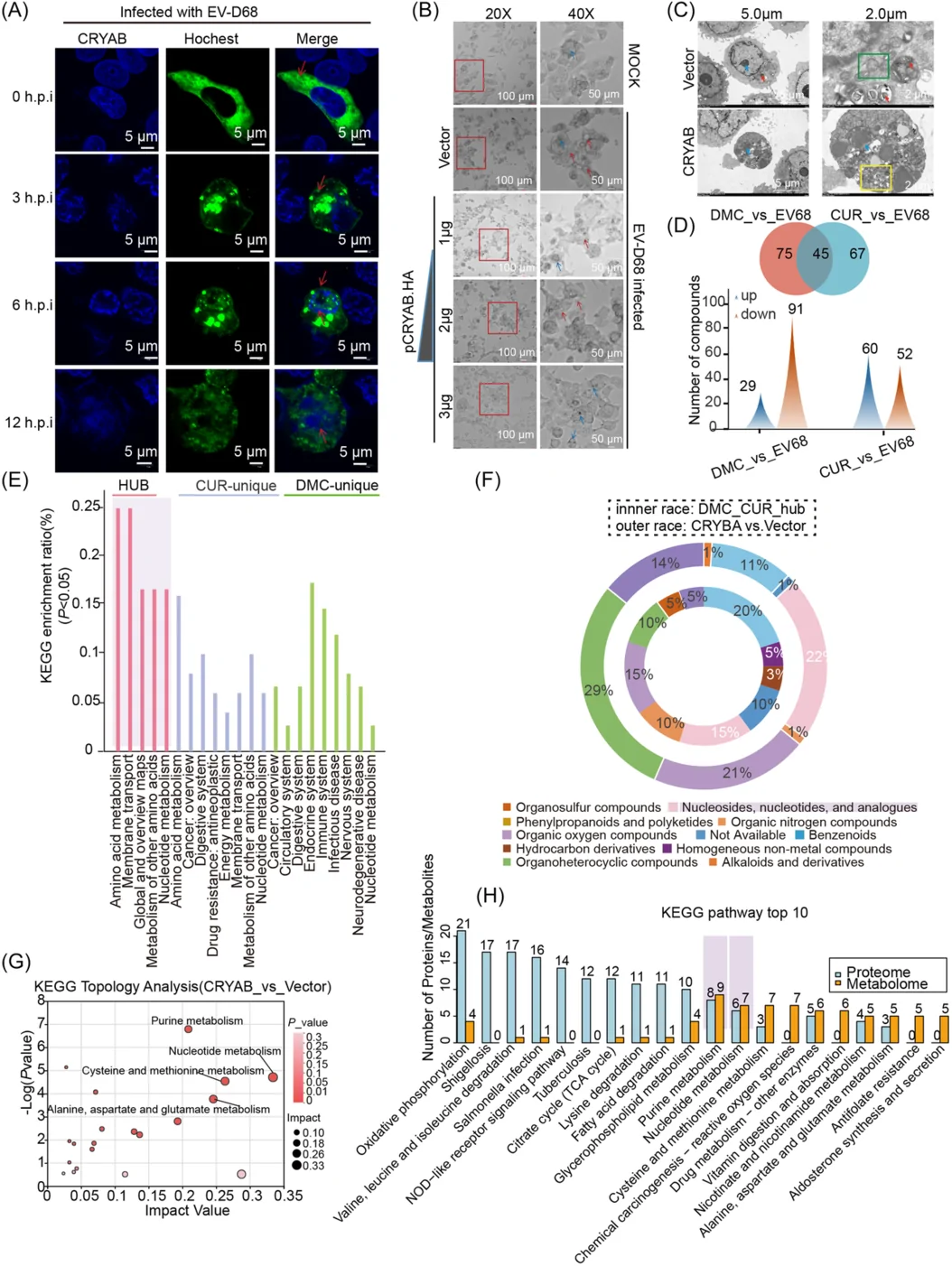
CRYAB translocation modulates the host response to EV‐D68 by regulating nucleotide metabolism. (A) IF analysis of CRYAB subcellular localization. RD cells (1 × 10⁵/well) seeded on coverslips in 24‐well plates were transfected with empty vector (2 μg/well) or CRYAB. HA (2 μg/well) for 48 h, followed by EV‐D68 infection. IF staining was performed at 0, 3, 6, and 12 hpi. CRYAB (green) and nuclei (blue) were visualized. (B) Morphological observation of cells. RD cells (5 × 10⁵/well) were transfected with empty vector (3 μg/well) or CRYAB. HA (1/2/3 μg/well) for 48 h was used to infect the cells with EV‐D68 (multiplicity of infection, MOI; MOI = 1). The cells were imaged via light microscopy at 18 hpi. Syncytial formation (red boxes), viral‐like vacuoles (red arrows), and nuclear abnormalities (blue arrows) are highlighted. (C) Transmission electron microscopy (TEM) analysis. RD cells (4 × 10⁶/dish) were transfected with empty vector (20 μg/dish) or CRYAB. HA (20 μg/dish) was added for 48 h, after which the mixture was infected with EV‐D68. The cells were fixed with glutaraldehyde at 18 hpi for TEM. Blue arrows: nucleoli; red arrows: cytoplasmic vesicles; green boxes: mitochondria; yellow boxes: large granular structures. (D) Venn diagram showing differentially abundant metabolites between the CUR‐ or DMC‐treated groups and the DMSO control group (VIP > 1, *p* < 0.05). Venn analysis was performed to identify overlapping and compound‐specific metabolic alterations. (E) KEGG pathway enrichment analysis of differentially abundant metabolites (DEMs). Metabolites with VIP > 1 and *p* < 0.05 were selected to identify those commonly or uniquely regulated by CUR and DMC compared with DMSO. (F) KEGG pathway enrichment of differentially abundant metabolites upon DMC/CUR treatment and CRYAB overexpression. The inner ring shows the percentage of enriched pathways among differentially abundant metabolites following DMC/CUR treatment; the outer ring shows that of CRYAB overexpression. The percentages represent the number of metabolites associated with each pathway divided by the total number of differentially abundant metabolites. (G) KEGG analysis of the effects of CRYAB overexpression. (H) Number of proteins/metabolic KEGG pathways (top 10, *p* < 0.05).

### CRYAB translocates to the nucleus and regulates nucleotide metabolism during EV‐D68 infection

Immunofluorescence revealed the intracellular redistribution of CRYAB during EV‐D68 infection. CRYAB shifted from diffuse in the cytoplasm (0 hpi) to aggregate (3 hpi), then partial nuclear translocation (6 hpi), and finally fully into the nucleus (12 hpi), with initial nuclear division (Figure 3A). These findings suggest that CRYAB may function through aggregation and potential nuclear interactions. No classical nuclear localization signal (NLS) sequence was identified by an online tool. Compared with the EV‐D68 infection group, treatment with an importin‐β inhibitor caused CRYAB to localize in the cytoplasm, preventing its entry into the nucleus (Figure S4A). The Co‐immunoprecipitation (Co‐IP) results also indicated that, following EV‐D68 infection, CRYAB strongly interacted with importin‐β, whereas no interaction was detected in the mock group (Figure S4B). In addition, upon dose‐dependent overexpression of CRYAB, cells presented a reduced cytopathic effect after EV‐D68 infection (red boxes), with decreased cytoplasmic vacuolation (red arrows) and decreased levels of cell death. Additionally, dark nuclear puncta, indicative of nuclear condensation, were observed (blue arrows) (Figure 3B). Transmission electron microscopy (TEM) revealed distinct ultrastructural differences. In vector‐transfected cells, the nuclei maintained intact nucleoli and nuclear membranes, and cytoplasmic virus‐like vesicles were encircled by mitochondria. Upon CRYAB overexpression, the nucleus divided, and granular structures accumulated perinucleously (Figure 3C).

To explore the mechanism underlying the nucleocytoplasmic transport of CRYAB, metabolomic profiling was performed after CUR and DMC treatment. Both compounds significantly altered amino acid and nucleotide metabolism pathways (*p* < 0.05) (Figure 3D,E). Comparative analysis of the differentially abundant metabolites revealed a high proportion of nucleic acid‐related metabolites in both treatment groups, with 22% in the drug‐treated group and 15% in the CRYAB overexpression group (Figure 3F). Similarly, compared with the vector control, CRYAB overexpression significantly modulated purine and nucleotide metabolism, and CRYAB overexpression upregulated antiviral‐related pathways and the cytosolic DNA‐sensing pathway (Figure 3G and Figure S5A–C). EggNOG functional enrichment analysis of CRYAB‐regulated differentially expressed proteins (DEPs) revealed that the upregulated DEPs were associated mainly with transcription, whereas the downregulated DEPs were enriched in energy production/conversion and lipid transport and metabolism (Figure S5D). Furthermore, integrative proteomic–metabolomic analysis of the CRYAB group confirmed enrichment of purine and nucleotide metabolism pathways, as identified by KEGG pathway analysis (Figure 3H).

### Multiomics reveals RBM26 as a putative effector in CRYAB‐driven remodeling of host protein networks during EV‐D68‐induced immune and metabolic responses

To elucidate the molecular mechanism by which CRYAB regulates nucleic acid metabolism, we performed integrated analyses combining proteomics, post‐translational modification (PTM) profiling, and immunoprecipitation‐mass spectrometry (IP‐MS) (Figure 4A). In total, 29 upregulated DEPs (termed hub‐up‐IP) that interact with CRYAB, including OAS1, IFNAR1, and IFIT1, and 16 downregulated DEPs (hub‐down‐IP), such as DHFR, VTN, and NT5DC2, were identified (Figure 4B).

**Figure 4:**
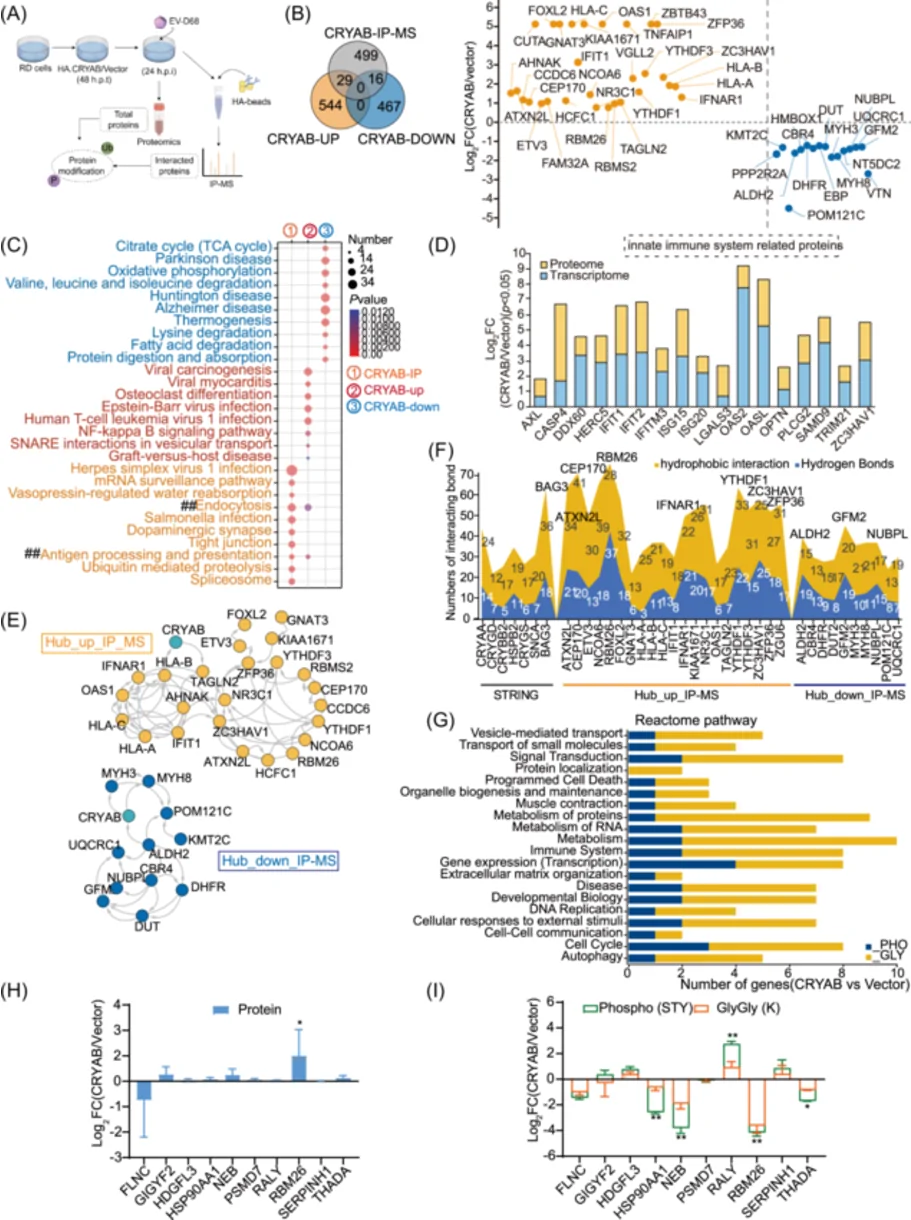
CRYAB‐regulated proteomic landscape and identification of downstream antiviral targets. (A) Experimental workflow for multiomics integration designed by Figdraw, including proteomics, PTM profiling, and IP‐MS. (B) Venn diagram and fold‐change statistics of the overlapping DEPs. Venn analysis identified common proteins among the IP‐MS‐related proteins, CRYAB‐upregulated DEPs, and CRYAB‐downregulated DEPs. The log_2_FC values (CRYAB vs. Vector) for these proteins are shown (*p* < 0.05, |log_2_FC| > 1.5). (C) KEGG enrichment analysis of CRYAB‐downregulated DEPs, CRYAB‐upregulated DEPs, and CRYAB‐interacting DEPs (*p < 0.05). ## Indicates pathways enriched in both CRYAB‐upregulated DEPs and CRYAB‐interacting DEPs. (D) Differential expression of innate immune‐related genes at the transcriptomic and proteomic levels. *p < 0.05, |log_2_FC| > 1.2 (log_2_FC = CRYAB‐overexpressing group vs. empty vector group). RD cells overexpressing CRYAB or empty vector for 48 h were infected with EV‐D68 (MOI = 1). The cells were harvested 24 h post infection (hpi) for transcriptomic and proteomic analyses. (E) Protein–protein interaction networks of the hub‐upCoIP (yellow) and hub‐downCoIP (blue) protein sets. (F) Number of interaction bonds (hydrophobic interactions, yellow; hydrogen bonds, blue) between CRYAB and protein clusters. (G) Reactome pathway analysis of PTM‐DEPs regulated by CRYAB. Phosphorylation (PHO, blue) and ubiquitination (GlyGly (K) modification, orange) are highlighted. (H) Overlapping DEPs between the global proteomic and PTM datasets, showing baseline expression fold changes (|log_2_FC| > 1.2). Significance: *p < 0.05; **p < 0.01. (I) Fold changes in posttranslational modification levels (|log_2_FC| > 1.2) for overlapping DEPs. Phosphorylation (phospho (STY), green) and ubiquitination (GlyGly (K), orange) are shown (*p < 0.05; **p < 0.01).

KEGG pathway enrichment revealed that the upregulated proteins and their interactors were predominantly involved in endocytosis and antigen processing/presentation pathways, whereas the downregulated proteins were enriched in metabolic pathways (Figure 4C). Further pathway analysis via iPTH 3.0 indicated that the downregulated proteins were associated mainly with lipid and energy metabolism, whereas the upregulated proteins and interactors were enriched in nucleic acid metabolism (Figure S6A).

Proteomic and transcriptomic profiling of EV‐D68‐infected RD cells revealed that CRYAB overexpression markedly upregulated the expression of innate immune genes (*OAS2*, *DDX60*, *IFIT1*, and *IFIT2*) compared with that in vector control cells (Figure 4D). Protein–protein interaction networks generated via STRING and visualized in Cytoscape highlighted complex associations within both the hub‐up‐IP and the hub‐down‐IP clusters (Figure 4E).

Molecular docking with ClusPro2.0 revealed that CRYAB exhibited stronger binding affinities with both protein clusters based on lower energy scores than interactions predicted from the STRING database references (Figure S6B). LigPlot+ analysis further revealed that CRYAB formed more extensive hydrogen bonds and hydrophobic interactions with its experimentally identified partners than with those predicted by STRING. Notably, RBM26 demonstrated high‐affinity binding to CRYAB, involving 28 residues in hydrophobic interactions and 37 in hydrogen bonding (Figure 4F and Figure S6C).

Reactome pathway enrichment of the PTM data revealed that phosphorylated and ubiquitinated proteins were enriched in pathways related to metabolism, protein homeostasis, immune responses, and RNA processing (Figure 4G and Figure S6D,E). Integration of the proteomics and PTM data indicated that CRYAB overexpression increased basal RBM26 protein levels (Figure 4H) while significantly reducing RBM26 phosphorylation and ubiquitination (Figure 4I), suggesting that CRYAB may stabilize RBM26 by suppressing its ubiquitin‐mediated degradation through direct binding.

### CRYAB stabilizes RBM26 by suppressing virus‐induced ubiquitination and cooperatively enhances antiviral immunity

Molecular dynamics simulations revealed that the CRYAB‐RBM26 complex underwent marked fluctuations during the first 30 ns but subsequently stabilized. The number of intermolecular hydrogen bonds remained consistently above 20 and increased to 80, reflecting strong and persistent interactions. The binding free energy, which was calculated via MM/GBSA, remained at approximately −145.93 kJ/mol, which is indicative of a stable and energetically favorable interface (Figure 5A; Table S1). Gibbs free energy landscape analysis corroborated this stability, revealing multiple low‐energy conformations with dense hydrogen bonding and complementary hydrophobic contacts. Docking studies further revealed that RBM26 engaged almost the entire surface of CRYAB, providing a structural basis for its high‐affinity, multistate interaction (Figure 5B).

**Figure 5:**
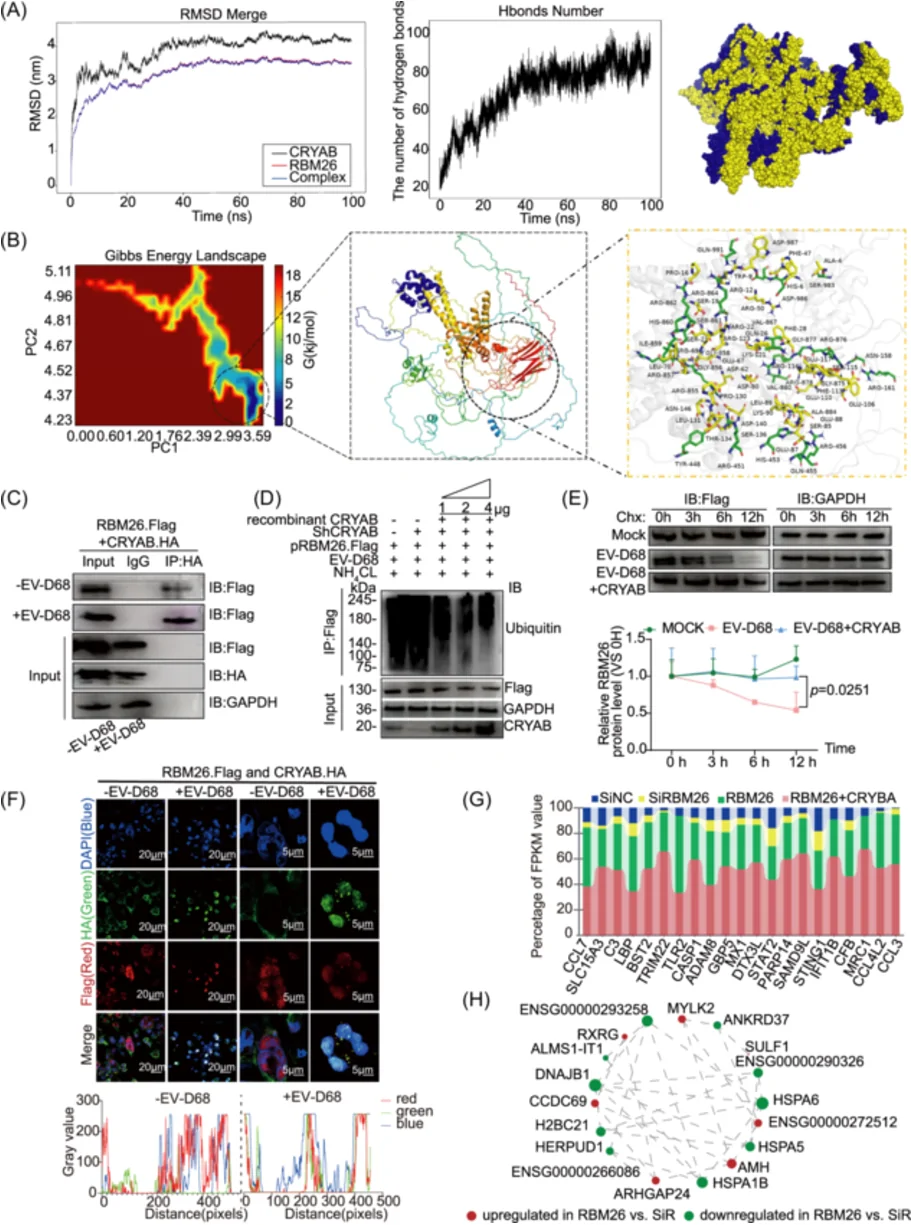
Identification of the interaction between CRYAB and RBM26. (A) RMSD analysis was performed to evaluate the stability of the protein–ligand complex. The RMSD trajectories of the protein (CRYAB, shown in black), the ligand (RBM26, red), and the CRYAB‐RBM26 complex (blue) were tracked. (B) Gibbs free energy landscape was plotted using the RMSD and radius of gyration (Rg) as collective variables to identify the conformational energy states of the complex. The blue regions represent low‐energy conformations, whereas the red regions indicate higher‐energy states. (C) Co‐immunoprecipitation (Co‐IP) confirmed the interaction between CRYAB and RBM26. RD cells were cotransfected with CRYAB and RBM26 and divided into two groups: one infected with EV‐D68 after 48 h and the other left uninfected. Total protein was extracted after 24 hpi and separated into three fractions: input, IgG control (incubated with IgG‐conjugated magnetic beads), and experimental (incubated with HA‐conjugated beads). The beads were washed to remove nonspecific proteins, followed by immunoblotting (IB) to detect target proteins. (D) Ubiquitination of RBM26 was performed by transfecting RD cells with a ShCRYAB plasmid, followed by EV‐D68 infection 48 h posttransfection. At 2 h post‐infection, the cells were incubated with media containing recombinant CRYAB at different concentrations (10, 20, and 40 µg/mL). The cells transfected with the control vector (SN) and without CRYAB supplementation served as blank controls. IP was used to measure the level of ubiquitinated RBM26. (E) A cycloheximide (Chx) chase assay was used to measure RBM26 degradation. RD cells were infected with EV‐D68, and after 2 h, the experimental group was incubated with medium supplemented with recombinant CRYAB (20 µg/mL). Three groups were set: MOCK (uninfected), EV‐D68, and EV‐D68 + CRYAB. At 0, 3, 6, and 12 h post‐Chx treatment (50 µg/mL), proteins were harvested, and RBM26 levels were assessed by Western blotting. Band intensities were quantified with ImageJ (normalized to *GAPDH*), and the fold change compared with that at 0 h was used for analysis via GraphPad Prism with Dunnett's multiple comparison test, *n* = 3, *p < 0.05. (F) Immunofluorescence analysis revealed the subcellular colocalization of CRYAB (green) and RBM26 (red) in the presence or absence of EV‐D68 infection. Colocalization was quantified under low‐magnification fields via ImageJ. (G) FPKM‐based gene expression analysis: The percentage of expression values (FPKM) for genes involved in innate antiviral immunity was calculated. Gene lists were obtained from the Human Protein Atlas (https://www.proteinatlas.org/), and data visualization was performed via the CNSknowledge platform (https://cnsknowall.com), a comprehensive bioinformatics tool. (H) Visualization of SiR_RBM26_hub gene expression correlation: Each node in the network represents a gene, with edges indicating expression correlation between genes or transcripts. The node size reflects the number of correlated interactions, where larger nodes indicate greater connectivity within the gene coexpression network.

Co‐IP confirmed that EV‐D68 infection strengthened the CRYAB‐RBM26 interaction (Figure 5C). Sh‐*CRYAB* increased RBM26 ubiquitination, whereas exogenous *CRYAB* reduced RBM26 ubiquitination in a dose‐dependent manner (Figure 5D). On the basis of the molecular docking results, a series of point mutation vectors were constructed according to the functional domain characteristics of CRYAB, and Co‐IP results revealed that the A1 and A2 mutants lost the ability to bind RBM26 and that these sites are located in the crystallin domain of CRYAB (Figure S7A,B). Next, the ubiquitination of RBM26 demonstrated that, compared with wild‐type CRYAB, the A1 and A2 mutants exhibited a weak inhibitory effect, whereas RBM26 maintained a low level of ubiquitination in the B1 and B2 groups (Figure S7C).

The sequencing results revealed that the specific ubiquitination sites of RBM26 were likely K529 and K538. We subsequently constructed RBM26 K‐site mutant plasmids, and the results revealed that both K529 and K538 were essential for RBM26 ubiquitination (Figure S7D). Given that K48‐linked polyubiquitination is a classic marker that mediates proteasomal degradation [36], our results showed that, compared with that of the wild‐type Ub plasmid, the RBM26 ubiquitination level in the Ub‐K48 mutant plasmid group was extremely low, similar to that in the blank control group, indicating that EV‐D68 infection‐induced RBM26 ubiquitination is dependent on K48‐linked polyubiquitination (Figure S7E). CHX chase assays demonstrated that CRYAB mitigated virus‐induced RBM26 degradation, thereby preserving RBM26 protein stability (Figure 5E). Notably, CRYAB translocated from the cytoplasm to the nucleus upon EV‐D68 infection, resulting in nearly complete nuclear colocalization with RBM26 (Figure 5F).

The overexpression of RBM26 significantly reduced the transcription level of the viral *2A* gene (Figure S8A). Transcriptomic analysis revealed that genes restored by *RBM26*, either alone or in combination with *CRYAB*, were significantly enriched in pathways related to cytokine signaling, interferon signaling, and the broader interferon response. Notably, the co‐expression of *RBM26* and *CRYAB* resulted in the most pronounced enrichment within immune‐related pathways. This included the cooperative upregulation of key antiviral effectors, such as *STAT2*, *GBP5*, *IFIT1B*, and *MX1* (Figure 5G and Figure S8B,C). Gene set variation analysis revealed enrichment of negative regulation of the viral cycle and antigen presentation in the co‐rescue group, whereas knockdown of RBM26 favored pathways linked to viral replication and impaired mRNA regulation (Figure S8D,E).

To further explore the target genes regulated by *RBM26*, we performed a Venn analysis, which identified 11 genes that were upregulated following *RBM26* silencing and downregulated upon *RBM26* re‐expression. Additionally, seven genes were found to be downregulated upon *RBM26* silencing and upregulated after *RBM26* re‐expression. These genes were found to form an interactive network, suggesting potential functional relationships (Figure 5H and Figure S8F). Collectively, these results demonstrate that CRYAB not only stabilizes RBM26 by inhibiting virus‐induced ubiquitination but also cooperatively amplifies RBM26‐driven transcriptional programs essential for innate antiviral immunity.

### CRYAB enhances alternative splicing reprogramming mediated by RBM26

*RBM26*, an RNA‐binding protein known to regulate mRNA splicing [37], has a widespread influence on alternative splicing (AS) events. The overexpression of RBM26 significantly increased global AS activity, predominantly through the induction of skipped exon (SE) events (Figure 6A). ΔPSI analysis revealed distinct splicing patterns: RBM26 overexpression promoted SE‐type shifts, whereas RBM26 knockdown predominantly affected alternative 5' splice site (A5SS) events. Notably, the combined overexpression of RBM26 and CRYAB (RBM26 + CRYAB group) elicited the most diverse AS repertoire, encompassing mutually exclusive exons and retained introns (RIs) (Figure 6B,C).

**Figure 6:**
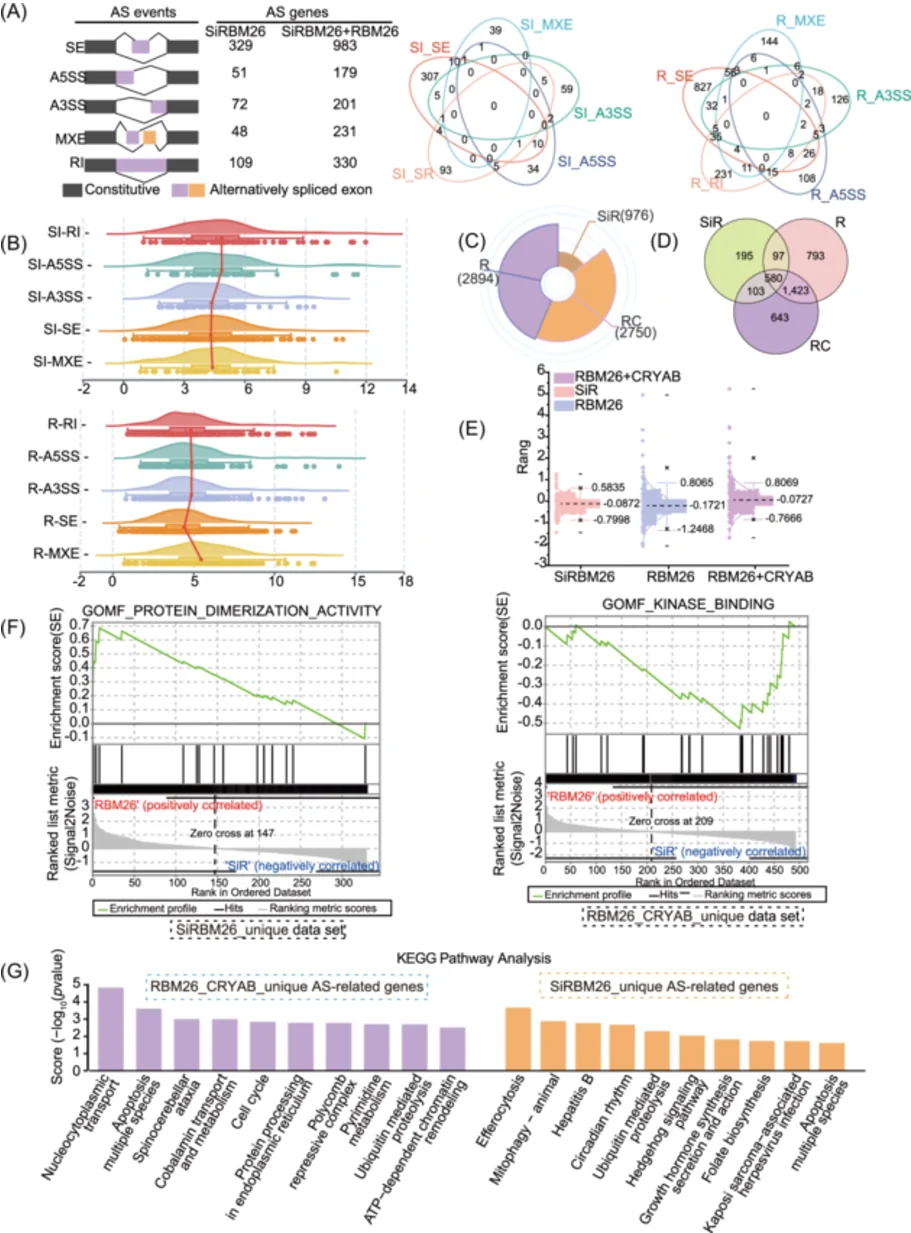
Transcriptomic analysis of RBM26‐mediated regulation of alternative splicing events. (A) Classification of alternative splicing (AS) events. Differential AS analysis was performed via rMATS (http://rnaseq-mats.sourceforge.net/index.html), which categorizes splicing events into five types: skipped exon (SE), alternative 5' splice site (A5SS), alternative 3' splice site (A3SS), mutually exclusive exons (MXE), and retained intron (RI). ΔPSI represents the difference in percent spliced‐in (PSI) values between two groups, calculated as ΔPSI = PSI_group1‐PSI_group2. A ΔPSI of 0 indicates no difference between groups; a ΔPSI > 0 indicates a greater likelihood of exon inclusion in group 1; and a ΔPSI < 0 indicates a greater likelihood of exon exclusion in group 1. The left panel shows the number of AS events for each splicing type, whereas the right panel presents a Venn diagram comparing AS genes in the SiRBM26 and RBM26 overexpression groups. (B) Raincloud plots of ΔPSI values for AS‐related genes in the SiRBM26 and RBM26‐overexpressing (SiRBM26 + RBM26) groups, generated via the CNSknowledge platform (https://cnsknowall.com), a comprehensive web‐based resource for data visualization and analysis. (C) Total number of AS‐related genes identified in the RBM26‐overexpressing (R), RBM26‐knockdown (SiR), and RBM26 + CRYAB‐overexpressing (RC) groups. (D) Venn diagram analysis of AS‐related genes across the R, SiR, and RC groups. (E) Box plot of FPKM values for AS‐related genes across the R, SiR, and RC groups, reflecting differences in expression level distribution. (F) Gene set enrichment analysis (GSEA). Genes were ranked by the Signal2Noise metric, and the enrichment score (ES) reflects the degree to which a gene set is overrepresented at the top or bottom of the ranked list. The normalized enrichment score (NES) is the standardized ES value, where a larger |NES| and smaller p‐adjust indicate greater enrichment significance. Only the top 20 gene sets according to |NES| are shown. The left panel represents enrichment results for the RBM26_CRYAB_unique AS‐related gene set, and the right panel represents enrichment results for the SiRBM26_unique AS‐related gene set. (G) KEGG pathway enrichment analysis. The top 10 enriched pathways are ranked by ascending *p‐*value. The left panel shows enrichment results for the RBM26_CRYAB_unique AS‐related gene set, and the right panel shows enrichment results for the SiRBM26_unique AS‐related gene set.

Comparative AS profiling revealed that 580 genes were commonly affected across groups, with 195 uniquely regulated genes in the *RBM26*‐knockdown group and 1423 unique events in the RBM26 + CRYAB co‐rescue group (Figure 6D). Expression profiling of these AS genes revealed increased dispersion and elevated mean expression in the RBM26 and RBM26 + CRYAB groups, with a hierarchical trend of RC > SiR > R (Figure 6E). Gene set enrichment analysis (GSEA) linked RBM26 knockdown‐specific genes to protein dimerization activity, whereas RBM26 + CRYAB‐specific AS genes were significantly enriched for kinase binding and phosphorylation‐associated pathways (Figure 6F). KEGG pathway analysis further revealed co‐rescue‐associated genes involved in nucleocytoplasmic transport, pyrimidine metabolism, and ubiquitin‐mediated proteolysis (Figure 6G).

Weighted gene co‐expression network analysis revealed distinct regulatory modules associated with *RBM26* knockdown versus co‐rescue conditions (Figure S9A). Pathway mapping of these modules indicated broader metabolic pathway engagement in the RBM26 + CRYAB group (Figure S9B). Moreover, integrated analysis of CRYAB‐associated proteomic and transcriptomic data revealed four key candidate targets, among which CMPK2 was consistently and significantly upregulated at both the transcript and protein levels (Figure S9C).

### Curcuminoids enhance mtDNA‐cGAS‐STING signaling to induce type I interferon production via RBM26 and modulate the effector phase through CRYAB

Alternative splicing analysis revealed that RBM26 re‐expression altered the splicing profile of cytidine/uridine monophosphate kinase 2 (*CMPK2*) compared with that in the siRNA control (SiR) group (Figure S10A). Among the six *CMPK2* transcript variants, ENST00000478738 presented the highest expression and was significantly upregulated in both RBM26‐reconstituted (SiR+RBM26) and RBM26 + CRYAB coreconstituted (SiR+RBM26 + CRYAB) cells, and total *CMPK2* mRNA levels paralleled these changes (Figure S10B,C). Furthermore, both DMC and CUR significantly upregulated the expression of *CMPK2* and *RBM26*. Silencing *CMPK2* or *RBM26* markedly inhibited the transcription of the viral *2A* gene (Figure S10D–F). These findings indicate that *CMPK2* is a downstream effector of RBM26‐mediated splicing regulation. *CMPK2* is a type I ISG that exerts antiviral effects. *CMPK2* encodes a mitochondrial nucleotide kinase that catalyzes the phosphorylation of cytidine and uridine to produce CDP and UDP [38]. Upregulation of *CMPK2* expression led to a decrease in cytidine and uridine levels (Figure 7A). Metabolomic analysis revealed that the overexpression of *CRYAB* or treatment with CUR/DMC significantly reduced the intracellular cytidine and uridine levels (Figure 7B), indicating that CRYAB and DMC/CUR promote increased nucleotide consumption. Next, we used high‐performance liquid chromatography (HPLC) to examine the effects of drug treatment and exogenous overexpression of RBM26 and CRYAB on the cytidine and uridine levels in cells. Compared with the control, EV‐D68 infection significantly increased cytidine levels. In contrast, compared with viral infection, DMC/CUR or RBM26/CRYAB overexpression markedly reduced cytidine levels (Figure 7C). No significant differences in uridine levels were detected between the groups (Figure S10G,H), suggesting that these treatments suppress virus‐induced cytidine upregulation.

**Figure 7:**
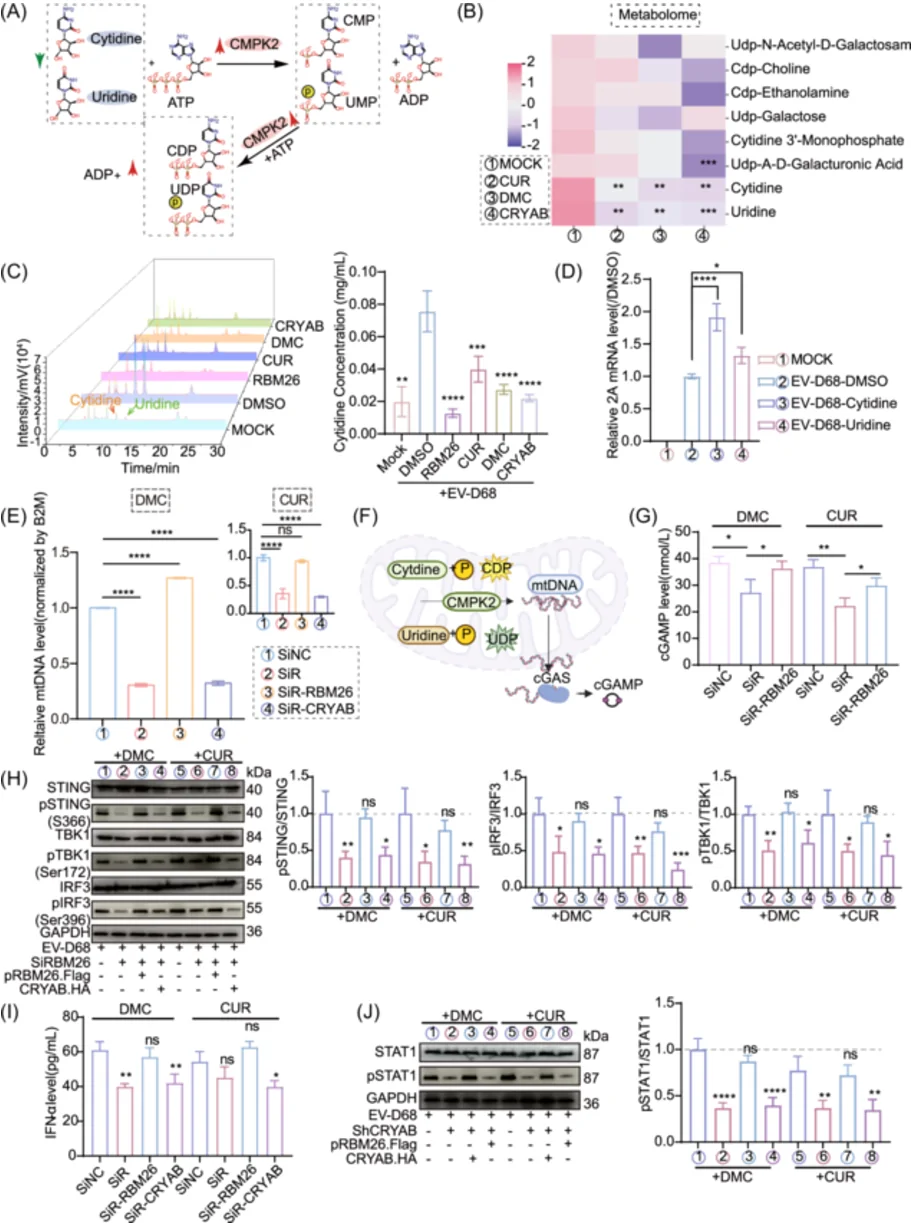
Identification of downstream targets of RBM26 and its regulatory mechanisms in nucleic acid metabolism. (A) Schematic of CMPK2‐mediated regulation. The upregulated elements are marked with red upwards arrows, and the downregulated elements are marked with green downwards arrows. (B) Heatmap of nucleic acid metabolism‐related metabolites (*n* = 3). Significant differences are indicated as follows: **p < 0.05; ***p < 0.001. (C) Intensities of cytidine and uridine. The high‐performance liquid chromatography (HPLC) profiles of cytidine and uridine in different experimental groups (left) and the statistical analysis of the cytidine content (right) are shown below. RD cells were seeded into six‐well plates and divided into six groups, with three replicates in each group. After cell adhesion, transfection treatments were performed: the mock group (untreated), DMSO group, CUR group, and DMC group were transfected with an empty vector; the RBM26 group was transfected with pRBM26. Flag plasmid (3 μg); the CRYAB group was transfected with pCRYAB. HA plasmid (3 μg). At 36 h posttransfection, the cells were cultured in serum‐free, antibiotic‐free medium and infected with the EV‐D68 virus (MOI = 1). 2 h after viral infection, the medium was replaced with normal culture medium. For the DMSO, RBM26, and CRYAB groups, DMSO was added to the medium, whereas the CUR and DMC groups received 40 μM and 20 μM drugs, respectively. After culture for 12 h, the cells were collected and subjected to sonication for 30 min. The cell supernatants were then collected for HPLC analysis to determine the cytidine and uridine contents. The data are shown as the mean ± SD (*n* = 3). Statistical analysis was performed via one‐way ANOVA: **p < 0.01; ***p < 0.001; ****p < 0.0001. (D) Relative mRNA level of the viral *2A* gene. RD cells in the logarithmic growth phase were seeded into six‐well plates at 80–90% confluency and divided into four groups. After cell attachment, the medium was replaced with serum‐ and antibiotic‐free medium. The mock group received no treatment, whereas the DMSO, cytidine, and uridine groups were infected with EV‐D68 (MOI = 1). Two hours post‐infection, all groups were switched to complete culture medium. Specifically, the DMSO group received medium containing DMSO; the cytidine group received medium supplemented with 15 μM cytidine; and the uridine group received 25 μM uridine. After 12 h of incubation, total RNA was extracted from the cells, reverse transcribed into cDNA, and analyzed via RT‐qPCR to quantify the expression level of the viral *2A* gene. *GAPDH* was used as the internal reference. The data are shown as the mean ± SD (*n* = 3). Statistical analysis was performed via one‐way ANOVA: *p < 0.05; ****p < 0.0001. (E) Relative mitochondrial DNA (mtDNA) levels. RD cells were seeded into 6‐well plates at 70–80% confluency and divided into eight groups. The cells were transfected with siRBM26 small RNA, while the control group was transfected with the siNC fragment. After 24 h, the medium was replaced with complete culture medium. The SiNC and SiR groups were transfected with an empty vector, whereas the SiR‐RBM26 group was transfected with pRBM26. Flag plasmid (3 μg), and the SiR‐CRYAB group with pCRYAB. HA plasmid (3 μg). After 24 h, the medium of the cells was changed to serum‐ and antibiotic‐free medium, and the cells were subsequently infected with EV‐D68 (MOI = 1) for 2 h. Following infection, one set of the SiNC, SiR, SiR‐RBM26, and SiR‐CRYAB groups was treated with complete medium containing 20 μM DMC, and the other set was treated with 40 μM CUR. After 12 h, total DNA was extracted, and the *ND1* copy number was quantified via qPCR. The mtDNA content was calculated on the basis of a standard curve, with B2M as the internal reference gene. The data are shown as the mean ± SD (*n* = 3). Statistical analysis was performed via one‐way ANOVA: *p < 0.05; **p < 0.01. (F) Schematic of the CMPK2‐mediated activation of mtDNA and cGAS, leading to the production of cGAMP. (G) cGAMP intensity. The SiNC, SiR, and SiR‐RBM26 groups from panel (E) were used. The supernatants from the cell lysates were collected, and the cGAMP levels were measured via ELISA. The data are presented as the mean ± SD (*n* = 3). Statistical significance was determined by one‐way ANOVA: *p < 0.05; **p < 0.01. (H) Western blots showing the STING‐IRF3‐TBK1 signaling pathway. The SiNC, SiR, SiR‐RBM26, and SiR‐CRYAB groups from panel (E) were used. The data are presented as the mean ± SD (*n* = 3). Statistical significance was determined by one‐way ANOVA: *p < 0.05; *p* < 0.01; ***p < 0.001; ns, not significant. (I) IFNα levels. The SiNC, SiR, SiR‐RBM26, and SiR‐CRYAB groups from panel (E) were used. Supernatants from the cell lysates were collected, and IFNα levels were measured via ELISA. The data are presented as the mean ± SD (*n* = 3). Statistical significance was determined by one‐way ANOVA: *p < 0.05; **p < 0.01; ns, not significant. (J) Western blots of STAT1. RD cells were seeded into six‐well plates at 70–80% confluency and divided into 8 groups. The cells were transfected with the shCRYAB plasmid (SiC), while the control group was transfected with the shNC vector (siNC). After 24 h, the medium was replaced with complete culture medium. The SiNC and SiC groups were transfected with an empty vector, whereas the SiC‐RBM26 group was transfected with pRBM26. Flag plasmid (3 μg), and the SiC‐CRYAB group with pCRYAB. HA plasmid (3 μg). After 24 h, the medium of the cells was changed to serum‐ and antibiotic‐free medium, and the cells were subsequently infected with EV‐D68 (MOI = 1) for 2 h. Following infection, one set of the SiNC, SiC, SiC‐RBM26, and SiC‐CRYAB groups was treated with complete medium containing 20 μM DMC, and the other set was treated with 40 μM CUR. The data are presented as the mean ± SD (*n* = 3). Statistical significance was determined by one‐way ANOVA: **p < 0.01; ****p < 0.0001; ns, not significant.

As a nucleotide component, cytidine is essential for viral RNA/DNA synthesis, genome replication, and protein production [39, 40]. Further analysis of the effects of cytidine and uridine treatments on EV‐D68‐infected RD cells revealed that cytidine significantly increased the transcription of the viral gene *2A* (*p* < 0.0001), whereas uridine slightly upregulated *2A* transcription (*p* < 0.05) (Figure 7D and Figure S10I), indicating that cytidine plays a more significant role in EV‐D68 replication than uridine does.

Additionally, *CMPK2* has been reported to regulate the mtDNA‐STING pathway [41]. To further investigate this, we quantified mtDNA levels via qPCR (Figure S10J). EV‐D68 infection suppressed mtDNA levels, whereas CUR or DMC treatment restored mtDNA levels (Figure S11A, left). Similarly, *RBM26* overexpression rescued mtDNA loss induced by siRNA‐mediated knockdown (SiR+RBM26 vs. SiR) (Figure S11A, right). Compared with the *RBM26*‐silenced group, DMC/CUR significantly increased mtDNA levels upon RBM26 plasmid re‐expression. The SiR and SiR‐CRYAB groups did not show any effect of DMC/CUR on mtDNA levels (Figure 7E). Knockdown of *CRYAB* led to a pronounced decrease in mtDNA levels, compared to the SiNC group. Notably, this defect was substantially rescued by the concomitant overexpression of *RBM26* (Figure S11B). These findings indicated that the induction of mtDNA by DMC/CUR depends on RBM26, not CRYAB.

When mtDNA leaks into the cytoplasm, it is recognized by cGAS, which binds to mtDNA and synthesizes cGAMP [42] (Figure 7F). We assessed cGAMP levels in cells and found that, under normal conditions, cGAMP levels were significantly increased by cGAS stimulation (vs. MOCK). DMC/CUR treatment reduced cGAMP levels (vs. DMSO), whereas in the RD‐EV‐D68 infection model, DMC/CUR increased cGAMP levels (Figure S11C). Furthermore, DMC/CUR‐induced cGAMP upregulation was dependent on RBM26 expression (Figure 7G).

Next, we examined the STING‐TBK1‐IRF3 signaling pathway. Both CUR and DMC inhibited this pathway in the absence of EV‐D68 infection but strongly activated it upon viral infection. RBM26 overexpression significantly upregulated STING and enhanced the phosphorylation of TBK1 and IRF3 (Figure S11D). Silencing *RBM26* led to a significant reduction in the DMC/CUR‐induced phosphorylation of STING, IRF3, and TBK1 in the STING pathway (Figure 7H), suggesting that DMC/CUR activation of the STING pathway is dependent on RBM26 expression.

The IFN‐I antiviral pathway involves the production and secretion of IFN‐I, its binding to receptors, and the activation of downstream ISGs [43, 44] (Figure S11E). We further examined the regulation of IFNα secretion by DMC/CUR and found that RBM26 overexpression significantly enhanced IFNα secretion. Co‐expression of CRYAB and RBM26 further increased IFNα secretion (Figure S11F). In contrast, silencing RBM26 significantly reduced the effects of DMC/CUR on IFNα secretion, whereas restoring RBM26 expression reversed this effect (Figure 7I). Multimodal omics data indicate that DMC/CUR, as well as exogenous overexpression of CRYAB or RBM26, also regulate the induction and effector phases of the IFN‐I response.

We then assessed the expression of proteins and genes involved in the IFN‐I effector phase. DMC/CUR treatment and CRYAB overexpression significantly increased STAT1 phosphorylation (Figure S11G). The regulation of STAT1 phosphorylation by DMC/CUR was dependent on CRYAB, as silencing CRYAB led to reduced STAT1 phosphorylation, which was restored upon re‐expression of the CRYAB plasmid (Figure 7J). Additionally, we measured the transcription levels of ISGs (*OAS1*, *MX1*, and *IFIT1*) during the IFN‐I effector phase. DMC/CUR regulation of ISG transcription was dependent on CRYAB, as silencing CRYAB resulted in the downregulation of ISG transcription, which was restored upon CRYAB plasmid re‐expression (Figure S11H). In the neonatal mouse model of EV‐D68 infection, DMC/CUR treatment significantly upregulated the transcriptional levels of *CRYAB*, *MX1*, *OAS1*, and other related genes in brain tissues (Figure S11I).

Together, these findings demonstrate that DMC/CUR modulates both the induction and effector phases of the IFN‐I pathway. The regulation of the induction phase depends on RBM26, whereas the regulation of the effector phase relies on CRYAB. CRYAB enhances the regulatory role of RBM26 in the IFN‐I induction phase.

## DISCUSSION

DMC and CUR, as active constituents of turmeric, are representative compounds in traditional Chinese medicine. They exhibit antiviral activity by interfering with the entry of viruses (such as human immunodeficiency virus, Zika virus, and dengue virus). The immunomodulatory and antiviral effects, inhibition of replication, regulation of immune responses, and antioxidant properties of CUR have been widely recognized [6, 45, 46]. Previous studies have demonstrated that CUR induces apoptosis in human hepatoma G2 cells via mitochondrial hyperpolarization and mtDNA damage [47] and that CUR can increase the mtDNA copy number in hepatocytes [48]. In this study, by leveraging network pharmacology analysis suitable for TCM [49], multiomics data mining [50], and molecular experimental validation, we found that during EV‐D68 viral infection, both CUR and DMC increased mtDNA levels by targeting the CRYAB‐RBM26‐CMPK2 axis, which subsequently activated IFN‐mediated antiviral responses and a broad spectrum of ISGs.

Our data identified CRYAB as a common downstream target of CUR and DMC, establishing a mechanistic link between natural compounds and host antiviral defense. CRYAB is conventionally known for its chaperone‐like functions and cytoprotective roles, which assist in protein folding and prevent aggregation [51, 52, 53, 54, 55]. Emerging evidence suggests that CRYAB is also closely involved in viral infection [56]. The stabilization of CRYAB suppressed EV71 replication in SH‐SY5Y (Human Neuroblastoma SH‐SY5Y cell line) cells [57], and virus infection induced CRYAB in the spleen and antigen‐presenting cell subsets in animal models [58]. Moreover, CRYAB facilitates the degradation of the hepatitis B virus X (HBx) protein, thereby inhibiting HBV gene expression and replication [53]. Our prior work also demonstrated that CRYAB stabilizes IFNAR1, thereby mitigating HBV‐mediated immune evasion [20].

Although the upregulation of CRYAB during cellular stress is well documented, its role as a regulatory scaffold in antiviral signaling remains incompletely defined. Through proteomic profiling and molecular docking, we found that CRYAB potentially contributed to the assembly and stabilization of key interferon signaling complexes, including IFITM3, OAS2, and DDX60. These observations support a novel role for CRYAB in antiviral immunity. Complementary metabolomic analyses revealed that CUR and DMC modulate nucleic acid metabolism, likely via CRYAB‐dependent mechanisms. Although curcuminoids have shown therapeutic potential in metabolic diseases such as metabolic syndrome, NAFLD, atherosclerosis, and diabetes [59], it remained unclear whether their antiviral effects involve CRYAB‐mediated regulation of nucleic acid metabolism.

By integrating proteomics, transcriptomics, posttranslational modification profiling, molecular docking, and dynamic simulation, we identified RBM26 as a downstream effector of CRYAB. RBM26 encodes a nuclear RNA‐binding protein involved in the process of nuclear RNA decay [60, 61]. Several RBM26 homologs have been implicated in innate immunity: RBM39 modulates ISGs expression [62], RBM10 is targeted by dengue virus to subvert host splicing and the immune response [63], and RBM24 serves as a host restriction factor against HBV [64]. Here, our results demonstrated that RBM26 distinctively regulated the AS of *CMPK2* and increased its transcription. *CMPK2* is an ISG with broad antiviral activity mediated through both immune regulation and direct viral inhibition [38, 65, 66]. Importantly, *CMPK2* encodes a mitochondrial nucleoside monophosphate kinase essential for the synthesis of deoxynucleotides required for mtDNA replication [67]. Elevated mtDNA levels activate the STING pathway, leading to type I IFN and ISG expression [42]. Our findings revealed that RBM26 notably increased mtDNA levels and activated the STING‐TBK1‐IRF3 pathway, leading to the secretion of IFN‐α and IFN‐β. In contrast, CRYAB alone elicited only modest type 1 interferon induction. Consistent with our previous results, in the HepG2‐NTCP‐HBV model, CRYAB did not initiate IFN production but instead stabilized IFNAR, enhancing downstream signaling [20]. These results highlight a context‐dependent role for CRYAB in IFN signaling, primarily through the modulation of downstream effectors.

In vitro, CUR and DMC activated the STING‐TBK1‐IFN pathway only in the context of viral infection. These observations align with previous reports, where CUR alleviated viral symptoms by suppressing TLR3/TBK1/IFN‐β signaling [68, 69], whereas curcumol inhibited IFN‐I release via direct STING signaling inhibition [70]. This context‐dependent regulation is particularly relevant given the dual role of STING in viral infections, canonically as a key mediator of antiviral defense, but also with noncanonical functions that can be hijacked by viruses [71]. EV‐D68 has been shown to co‐opt STING to facilitate the formation of replication organelles and regulate immunometabolism, highlighting a strategy by which the virus subverts the antiviral potential of STING for its own replication. Consistent with this, other enteroviruses (e.g., EV‐A71 and CV‐A16) also target STING signaling [72]. Our data support these observations by demonstrating that EV‐D68 infection potently inhibits the STING‐TBK1 signaling axis and that CUR/DMC treatment effectively antagonizes this viral suppression, restoring STING‐dependent IFN signaling.

Our study reveals the CRYAB‐RBM26 axis as a previously unrecognized regulatory hub that modulates innate antiviral immunity by fine‐tuning the cGAS‐STING‐interferon (IFN) pathway. The identification of the CRYAB‐RBM26 axis not only enriches the molecular network of innate immune regulation but also provides a novel therapeutic candidate for antiviral intervention, with unique advantages in balancing immune defense and homeostasis, laying a foundation for subsequent translational exploration.

A critical concern is whether chronic targeting of the CRYAB‐RBM26 axis is associated with autoimmunity. The host has evolved multilayered ubiquitin‐dependent degradation pathways to terminate IFN‐I signaling and maintain homeostasis, acting as “brakes” to prevent excessive activation even when the CRYAB‐RBM26 axis is targeted. After the IFN response, IFNAR1 is ubiquitinated and degraded via lysosomes, attenuating JAK‐STAT signaling [73]. Activated STING is degraded through RNF213‐mediated lysosomal or TRIM29‐mediated proteasomal degradation to halt downstream signaling [74, 75, 76]. cGAS is degraded by RNF185 (proteasome) [77], limiting excessive IFN production. These conserved pathways form a self‐limiting loop: the CRYAB‐RBM26 axis enhances immunity to clear viruses, while degradation mechanisms quickly terminate excess IFN signaling, preventing prolonged pathway activation. We subsequently explored the outcome of the IFN pathway during late‐stage activation of the CRYAB‐RBM26 axis.

Phosphorylation of CRYAB plays a pivotal role in post‐myocardial infarction ventricular remodeling [78], and its interaction with filamentous proteins implies a potential involvement in the regulation of mechanical properties. The synergistic regulatory mode between biological factors and mechanical properties has long been a research hotspot in the modulation of vascular pathologies, with various mechanical detection approaches acting as a critical bridge for investigating biological targets and their underlying mechanical mechanisms [79, 80, 81]. Furthermore, recent studies have demonstrated that many natural small‐molecule compounds exert multi‐faceted regulatory effects on vascular mechanical properties; for example, procyanidins can restore the residual stress of decellularized blood vessels via multi‐scale mechanical regulation [82, 83]. As a class of natural small‐molecule compounds, curcuminoids that target CRYAB to modulate the biomechanical axis hold substantial research potential in the context of cardiovascular diseases. However, future studies need to clarify the safety profile, therapeutic window, and disease specificity of CRYAB‐RBM26‐mediated immunomodulation in cardiovascular diseases via adopt chronic activation models and biomechanical approaches.

While this study focuses on mechanistic insights, our findings offer meaningful translational potential. Curcuminoids have shown favorable safety profiles in prior human trials for other indications, with tolerated doses of up to several grams per day [84, 85], supporting their repurposing potential. The identification of CRYAB and RBM26 as host targets also provides a resistance‐resilient strategy for antiviral intervention. Moreover, our data demonstrate that DMC exerts greater potency at lower doses in a dose‐dependent manner. This enhanced efficacy may be directly attributed to its improved physicochemical properties [17, 18], particularly its superior stability and increased water solubility at room temperature, compared to CUR under physiological conditions. These advantageous properties, as reported in prior studies, stem from the absence of a methoxy group on DMC's aromatic ring [15, 16]. Future efforts will focus on evaluating the bioavailability of DMC in advanced formulations and monitoring host immune activation to ensure therapeutic efficacy without adverse inflammatory responses.

Several key questions remain to be addressed. The upstream mechanism by which CUR and DMC upregulate CRYAB is not yet defined, nor is the mechanism of CRYAB nuclear translocation. While this study focused on the shared antiviral mechanisms of CUR and DMC, the compound‐specific differences in their antiviral activities remain to be elucidated. Future studies should aim to identify distinct molecular targets and signaling pathways regulated individually by CUR and DMC to facilitate more refined, personalized therapeutic strategies. Further investigations are warranted to determine the therapeutic window and safety implications of targeting CRYAB in viral versus tumor settings. To elucidate the precise mechanism by which RBM26 regulates *CMPK2* alternative splicing, future work will employ ChIP‐seq and RIP‐seq approaches to map RBM26 binding sites and their splicing regulatory network with increased resolution.

## CONCLUSION

In summary, we identified a novel CRYAB‐RBM26‐CMPK2 axis regulated by CUR and DMC that connects host nucleic acid metabolism to interferon signaling. These findings reveal a previously unrecognized mechanism by which natural compounds enhance antiviral responses and provide a foundation for the development of CRYAB‐centered host‐targeted therapeutic strategies. Notably, we report for the first time that RBM26 promotes mtDNA accumulation, establishing a mechanistic link between RNA‐binding proteins, mitochondrial metabolism, and antiviral immunity.

## METHODS

### Cells and virus

RD cells (ATCC: CCL‐136) were cultured in DMEM (Gibco, 11995) supplemented with 10% FBS and 1% penicillin–streptomycin (Gibco, 15140) at 37°C in 5% CO_2_. At 80% confluence, the cells were infected with EV‐D68 at an MOI of 1. The virus, kindly provided by Prof. Mingzhou Chen (Hubei University), was harvested upon 90% cytopathic effect, clarified by centrifugation and filtration, and titrated via a plaque assay for subsequent use.

### Plasmids and reagents

HA‐tagged CRYAB and Flag‐tagged RBM26 cDNAs were subcloned and inserted into pcDNA3.1/Zeo(+) (GenScript) via the XhoI and XbaI restriction sites. These constructs were subsequently transfected into RD cells with QuickShuttle‐Enhanced reagent (Biodragon, KX0110042). The following antibodies were used in this study: anti‐EV‐D68 VP1 (GTX132312, GeneTex), anti‐GAPDH (A01622), anti‐HA (51064‐2‐AP), anti‐Flag (20543‐1‐AP), and anti‐RBM26 (16823‐1‐AP) from Proteintech; and anti‐TBK1 (T55145), anti‐pTBK1 (TA8190), fluorescent secondary (Alexa Fluor 488, 568, and 633), and HRP‐conjugated goat anti‐mouse/rabbit antibodies from Abmart. The following antibodies were obtained from HUABIO: anti‐STAT1 (ET1606–39), anti‐pSTAT1 (ET1611–20), anti‐STING (HA722832), anti‐pSTING (HA723137), anti‐IRF3 (ET1612–14), and anti‐pIRF3 (ET1608–22). Magnetic beads for HA, Flag, protein A/G magnetic beads (HY‐K0202), curcumin (HY‐N0005), demethoxycurcumin (HY‐N0006), cytidine (HY‐B0158), uridine (HY‐B1449), poly(I:C) (HY‐107202), and poly (dA:dT) (HY‐138646) were obtained from MCE. IP lysis buffer was obtained from Beyotime. ELISA kits for IL‐6, IL‐1β, TNF‐α, IFN‐α (EHC144a.48), IFN‐β (EHC026b.48), and cGAMP (501700–96) were obtained from NeoBiosience. All primary antibodies and secondary antibodies were diluted at 1:500 and 1:5000, respectively. The primer sequences are listed in Table S2.

### Mouse infection and treatment

The institutional ethics committee approved the animal procedures. Based on ProTox 3.0 (LD50: 2000 mg/kg), neonatal BALB/c mice (<24 h old, *n* = 6/group) were assigned to high (500 mg/kg), medium (250 mg/kg), or low (125 mg/kg) dose groups. DMC and CUR were dissolved in 5% DMSO and administered intraperitoneally. The mice were intracranially injected with EV‐D68 (1 × 10^7^ PFU), and then drug treatments were given at 6, 12, 24, and 36 h postinfection, twice daily for 7 days. Clinical scores (0–5) and body weights were monitored daily. On Day 14, the mice were euthanized, and the brain, lung, liver, and spleen were collected for H&E, RT‐qPCR, or ELISA.

### Histological analysis (H&E staining)

Brains, lungs, livers, and spleens were collected from decapitated mice and fixed in 4% paraformaldehyde for 10 min. Tissues were paraffin‐embedded and sectioned at 4 μm. The sections were deparaffinized in xylene (3×), rehydrated through a graded ethanol series (100%, 95%, 70%), and rinsed in tap water. Hematoxylin staining was performed for 3 min, followed by a 5‐min water rinse and a 2‐min eosin counterstain. The slides were dehydrated, cleared in xylene, and mounted with neutral resin. Imaging was performed using a PerkinElmer Vectra Polaris, and four representative sections per organ were analyzed.

### Enzyme‐linked immunosorbent assay (ELISA)

The brain and lung tissues were homogenized on ice, and the supernatants were used to measure the IL‐6, IL‐1β, and TNF‐α levels via ELISA according to the manufacturer's instructions. For the cell‐based assays, cGAMP, IFNα, and IFNβ were detected via an ELISA kit, and the absorbance at 450 nm was measured via SoftMax Pro v5.

### Drug treatments

RD cells were treated with DMC or CUR (0–40 μM) for 24 h, and viability was assessed via a CCK‐8 assay. For infection assays, cells were infected with EV‐D68 (MOI = 1) for 2 h and then treated with DMC or CUR for 24 h. Viral *2A* gene expression was analyzed via RT‐qPCR. For transcriptome analysis, EV‐D68‐infected RD cells or HBV‐infected HepG2 cells were treated with DMC or CUR (20 μM) for 24 h before RNA‐seq to assess CRYAB transcription. In uninfected RD cells, DMC (20 μM) or CUR (40 μM) was applied for 48 h for RT‐qPCR and immunofluorescence to assess CRYAB mRNA and protein localization. For CRYAB knockdown, RD cells were transfected with shCRYAB for 48 h, infected with EV‐D68, and treated with DMC or CUR (20 μM). EV‐D68‐infected RD cells were treated with DMC or CUR (20 μM) for 18 h. Supernatants were collected, and IFN‐α/β levels were measured via ELISA kits (Yamei Biotech). Poly(I:C) (30 nM/well) served as a positive control.

### Transfection and gene knockdown

The cells were seeded into appropriate culture plates or dishes and transfected once they reached 70–80% confluency. For gene overexpression and CRYAB knockdown via shRNA vectors, transfections were performed with enhanced transfection reagent (KX0110042, Bioaolong) following the manufacturer's instructions. Briefly, the transfection reagent and plasmid DNA were each prediluted in normal saline and then mixed and added directly to cells maintained in complete medium without the need for medium replacement.

For siRNA‐mediated knockdown (SiR), the BioShuttle siRNA/miRNA transfection reagent (KX0110049, Bioaolong) was used. The siRNA and reagent were mixed at a mass‐to‐volume ratio of 1:2. Prior to transfection, the culture medium was replaced with serum‐free medium to facilitate uptake.

### RNA sequencing and data analysis

In our previous study, multiomics sequencing was performed to explore the antiviral mechanism of DMC/CUR [86]. Total RNA was extracted via the TRIzol® reagent (Thermo Fisher Scientific, USA, Cat No. 15596026) and subsequently purified via the RNeasy Mini Kit (Qiagen, Germany, Cat No. 74104). The quantitative assessment of RNA was conducted via a 5300 Bioanalyzer (Agilent) and ND‐2000 (NanoDrop Technologies). Fragmented RNA was then employed for cDNA library construction and sequencing, which were performed by Shanghai Majorbio Biopharm Technology Co., Ltd. on the Illumina NovaSeq 6000 platform (Illumina). Differentially expressed genes (DEGs) were analyzed with DESeq2 and DEGseq, with thresholds of |log_2_FC| ≥ 1.5 and *p*‐value_adjust ≤ 0.05 (DESeq2). GO functional enrichment and KEGG pathway analyses of the DEGs were conducted via Goatools and KOBAS [87], with significance defined by Bonferroni‐adjusted *p* values ≤ 0.05. Various plots were generated via R's ggplot2 v3.3.5 and GraphPad Prism v8.4.3, and heatmaps were produced with TBtools [88]. The original RNA sequencing data were uploaded to NCBI GEO, which is accessible at https://www.ncbi.nlm.nih.gov/sra/PRJNA1298549.

### Metabolite extraction and analysis

The cell metabolites were extracted with precooled methanol: water (80:20, v/v) containing 0.02 mg/mL L‐2‐chlorophenylalanine. After vortexing and sonication in an ice‐water bath, the samples were incubated at −20°C for 1 h and centrifuged (12,000 *g*, 15 min, 4°C), and the supernatant was vacuum‐dried. The dried extracts were reconstituted in acetonitrile: water (1:1, v/v), sonicated, centrifuged again, and analyzed via LC‐MS/MS [89]. Metabolites were annotated via HMDB, Metlin, and MJDB. The data were processed on the Majorbio Cloud platform [90].

### Proteomic analysis

Total proteins were extracted from RD cells via phenylmethylsulfonyl fluoride‐supplemented (PMSF‐supplemented) RIPA buffer, digested with trypsin, desalted, and vacuum‐dried. Peptides were quantified (Thermo, Cat No. 23275), separated via UPLC (Vanquish Neo, Thermo), and analyzed via an Orbitrap Astral mass spectrometer in DIA mode (m/z 100–1700, 1.5 kV, positive ion mode). Protein and PTM identification were performed via MaxQuant. Bioinformatic analyses, including Reactome pathway enrichment and STRING protein–protein interaction network construction, were conducted via the Majorbio Cloud and Cytoscape. Heatmaps were generated via TBtools. Proteomic data are available at proteomeXchange (PXD066985).

### Cell viability assay

The CCK‐8 assay was used to evaluate the effect of the drug on cell activity as previously reported [91, 92]. The IC_50_ values were calculated via GraphPad Prism 8.0. Briefly, logarithmically growing cells were seeded into 96‐well plates, and after adhering, the medium was replaced with fresh culture medium. The cells were then treated with various concentrations of the drug for 36 to 48 h. Following treatment, cell viability was measured according to the manufacturer's instructions, with the optical density determined at a wavelength of 450 nm.

### Reverse transcription‐quantitative PCR (RT‐qPCR) and quantitative PCR (qPCR)

RNA and DNA extraction, as well as qPCR procedures, were performed as previously described in our study [20]. Briefly, total RNA was extracted via TRIzol Reagent (Invitrogen), followed by DNase treatment to eliminate genomic DNA contamination and subsequent cDNA synthesis via a commercial kit (Promega). The synthesized cDNA samples were stored at 4°C and then used as templates for RT‐qPCR analysis to quantify the relative expression levels of target genes, with *GAPDH* used as the reference sample. qPCR was then conducted to quantify relative mRNA levels. Genomic DNA was extracted from the lysed cells via the Qiagen QIAamp DNA Mini Kit (Qiagen) and quantified via qPCR. A SYBR Premix Ex Taq II Kit (Takara) was used according to the manufacturer's protocol. The specific primer sequences are listed in Table S2.

### Mitochondrial DNA/Nuclear DNA copy number quantification by qPCR

RD cells were seeded in six‐well plates and divided into mock, DMSO, CUR, DMC, siR, and SiR‐R groups. The mock group served as a control, while the other groups were virus‐treated. The CUR and DMC groups were treated with drugs for 6 h before viral infection, and the siR and SiR‐R groups were transfected for 48 h before infection with EV‐D68 (MOI = 1). After 18 h of infection, total DNA (including mtDNA and nuclear DNA) was extracted via a Tiangen DNA extraction kit (DP304, Tiangen Biotech). The extracted DNA was used as a template for qPCR to quantify DNA copy numbers. Amplification was performed according to the manufacturer's instructions. Primers for mtDNA (*ND1*) and nuclear DNA (*B2M*) were used as previously described [93]. A standard curve was generated using mtDNA standards. ΔCt values (ND1‐B2M) were normalized, and copy numbers were calculated by comparing the ΔCt values to the standard curve. The primer sequences are listed in Table S2. All experiments were repeated independently three times.

### Western blotting

RD cells were transfected with CRYAB plasmids (1–4 μg/well) and infected with EV‐D68. Then, WB was performed as previously described in our study [94, 95, 96, 97, 98]. Briefly, after lysis with PMSF‐containing RIPA buffer, the supernatants were collected after centrifugation. Proteins were denatured, resolved by 10% SDS‐PAGE and transferred to PVDF membranes. After blocking, the membranes were incubated with primary (1:500) and HRP‐conjugated secondary antibodies (1:5000), followed by enhanced chemiluminescence detection. The signal intensity was analyzed via ImageJ. The experiments were performed in triplicate.

### Ubiquitination assay

Ubiquitination assays were conducted as described previously [20]. RD cells were cotransfected with pRBM26. Flag plasmids and pCRYAB. HA for 48 h, followed by treatment with 10 μM NH_4_Cl for 8 h. The cells were then lysed in IP buffer at 4°C for 30 min. The lysates were incubated overnight with anti‐Flag antibody, followed by incubation with A/G agarose beads for 12 h at 4°C. After the complexes were washed four times with ubiquitination buffer, the beads were eluted and analyzed by 10% SDS‐PAGE.

### Coimmunoprecipitation (Co‐IP) and LC‐MS/MS analysis

RD cells were cotransfected with pRBM26.Flag and pCRYAB.HA plasmids via the QuickShuttle‐Enhanced reagent (Biodragon). After 48 h, the cells were infected with EV‐D68 and harvested 24 h later. The cells were lysed in IP buffer (Beyotime) supplemented with PMSF (Roche), incubated on ice for 30 min, and centrifuged at 14,000 *g* for 30 min at 4°C. The supernatants were divided as follows: 100 μL was reserved as input for Western blotting; the remainder was incubated overnight at 4°C with either HA or IgG magnetic beads. The immunoprecipitates were washed with TBS‐T (0.1% Tween‐20) and then analyzed by Western blotting or subjected to LC‐MS/MS (Tsingke). For protein identification, MaxQuant was used, with carbamidomethylation as a fixed modification and phosphorylation/ubiquitination as variable modifications (max. 2 missed cleavages). Protein interaction networks were generated via STRING and visualized via Cytoscape.

### Molecular docking and dynamics

CRYAB‐interacting targets were predicted via IP‐MS and modeled via AlphaFold2. Docking with CRYAB was performed on ClusPro, and complexes were visualized via LigPlot+. The binding frequency was summarized in Excel. MD simulations were conducted in GROMACS 2022.3 via the AMBER99SB‐ILDN force field and TIP3P water. Ligand parameters were prepared with GAFF (AmberTools22) and RESP charges (Gaussian 16 W). After energy minimization, the systems underwent 100 ps NVT and NPT equilibration (300 K, 1 bar), followed by 100 ns production runs (2 fs timestep). The analyses included the RMSD, RMSF, radius of gyration, and MM/GBSA energy. Structures and trajectories were visualized in PyMOL.

### HPLC analysis of the cytidine and uridine contents

The cellular concentrations of cytidine and uridine were determined via HPLC. Standard curves for both nucleosides were prepared by injecting known concentrations of cytidine and uridine, and the respective contents in the experimental groups were calculated on the basis of these curves. Chromatographic separation was achieved via a ZORBAX SB‐Aq column (250 mm × 4.6 mm, 5.0 μm). The mobile phase consisted of a gradient mixture of methanol and water. The gradient program was as follows: 0–10 min, 96% water; 10.01–15 min, 94.5–93% water; and 15.01–20 min, 93–88% water. The flow rate was set at 0.5 mL/min, and the column temperature was maintained at 30°C. The absorbance was monitored at 260 nm, and a sample volume of 10 μL was injected for each analysis.

### Statistical analysis

Statistical analyses were conducted via GraphPad Prism 8.0. Normality of continuous variables was verified individually via the Shapiro–Wilk test. The data are presented as the mean ± SD (standard deviation) from three biological replicates of at least three independent experiments. Prior to the parametric analyses, the normality of all the quantitative datasets was evaluated via the Shapiro–Wilk test (*p* > 0.05). Comparisons between two groups were made via Student's *t*‐test; comparisons among multiple groups were performed via one‐way ANOVA with Tukey's post hoc test (one factor). The Friedman test was used for nonparametric overall difference analysis (Shapiro–Wilk test: *p* < 0.05); significant results prompted pairwise comparisons via the Wilcoxon rank‐sum test with Bonferroni correction. Survival analyses were performed via the log‐rank test. In both proteomic and post‐translational modification datasets, contaminant and reverse database entries (e.g., from GlyGly (K)Sites.txt, Phospho (STY)Sites.txt, and proteinGroups.txt) were removed. Data were log_2_‐transformed and normalized using quantile normalization. Differentially expressed proteins (DEPs) were identified using the R package t.test, with thresholds set at fold change (FC) > 1.2 or <0.83 and *p* < 0.05. To minimize technical variation from sample preparation and instrumentation, peak intensities were normalized using sum normalization. Variables with a relative standard deviation > 30% in QC samples were excluded. The resulting matrix was log_10_‐transformed for downstream analyses. For transcriptomics, gene expression levels were quantified by RSEM and normalized using TPM. Differential expression analysis was performed using both DESeq2 and DEGseq. Genes with |log_2_FC | ≥ 1 and FDR ≤ 0.05 (DESeq2) or FDR ≤ 0.001(DEGseq) were considered differentially expressed. Alternative splicing events were identified using the rMATS algorithm. Statistical significance was defined as *p* < 0.05, with significance denoted as ns, *p* > 0.05, *p <  0.05, **p < 0.01, ***p < 0.001, and ****p < 0.0001.

## AUTHOR CONTRIBUTIONS

**Tingting Chen** and **Xiang Wang**: Writing—review and editing; writing—original draft; methodology; investigation. **Lu Wang** and **Jun Hao Wan**: Methodology; investigation; data curation; conceptualization. **Xinyue Fan, Siqin Meng, Zhixing Jin,** and **Yuzhang Liang**: Methodology; software; project administration. **Guangyuan Zhang**: Writing—review and editing; resources, methodology, investigation. **Tingting Chen** and **Guangchao Zang**: Methodology; funding acquisition; formal analysis; conceptualization. **Li Qin, Lirong Tan,** and **Anna Malashicheva:** Writing—review and editing; validation. **Guixue Wang** and **Yu Du**: Resources; supervision; funding acquisition, conceptualization, writing—review and editing. **Haoze Li, Ziyuan Wan, Xiaotong Xu,** and **Yan Zhang**: Visualization; investigation; methodology; data curation. All authors have read the final manuscript and approved it for publication.

## CONFLICT OF INTEREST STATEMENT

The authors declare no conflicts of interest.

## ETHICS STATEMENT

The ethics application of animal studies (No. IACUC‐CQMU‐2024‐0669) was approved by the Animal Ethics Committee of Chongqing Medical University. All efforts were made to minimize the suffering of the animals and reduce the number of animals used.

## Supporting information


**Figure S1.** Antiviral efficacy of CUR and DMC in the EV‐D68 mouse model.
**Figure S2.** Upstream regulators of CRYAB modulated by CUR/DMC.
**Figure S3.** Differential gene expression and molecular docking analysis in the HepG2‐HBV infection model.
**Figure S4.** CRYAB subcellular localization and interaction with importin β during EV‐D68 infection.
**Figure S5.** Multi‐omics analysis of the downstream signal pathway targeted by CRYAB.
**Figure S6.** Molecular docking analysis of CRYAB‐interacting proteins and PTM‐related protein alterations.
**Figure S7.** Identification of the interaction between CRYAB and RBM26.
**Figure S8.** Investigating the contribution of CRYAB to RBM26.
**Figure S9.** WGCNA‐based identification of trait‐associated gene modules.
**Figure S10.** Identification of downstream targets of RBM26 and its regulatory mechanisms in the IFN‐I pathway.
**Figure S11.** RBM26 deficiency alters nucleotide metabolism and antiviral signaling, impairing the metabolic support for EV‐D68 replication.

**Table S1.** Delta (Complex–Receptor–Ligand)*. Calculate the binding energy and interactions between the ligand and protein using MM/GBSA. The units are in kcal/mol.
**Table S2.** The primers and oligonucleotides used in this paper.

## Data Availability

Proteomic data have been deposited in the ProteomeXchange Consortium via the PRIDE partner repository with the dataset identifier PXD066985 (https://proteomecentral.proteomexchange.org/cgi/GetDataset?ID=PXD066985). The RNA sequencing data are available from the NCBI Gene Expression Omnibus under accession number PRJNA1298549 (https://www.ncbi.nlm.nih.gov/sra/PRJNA1298549). The new code has been updated to GitHub (https://github.com/Ctt9954/Curcuminoids-Amplify-Host-Innate-Antiviral-Immunity-via-the-CRYAB-RBM26-Axis-in-Viral-Infection). Supplementary materials (figures, tables, graphical abstract, slides, videos, Chinese translated version, and update materials) may be found in the online DOI or iMeta Science http://www.imeta.science/.
