# Supplementary material for: Curcuminoids amplify host innate antiviral immunity via the CRYAB‐RBM26 axis in viral infection
Source: Imeta. 2026 Feb 24;5(1):e70111. doi: 10.1002/imt2.70111 (PMC13726585; doi:10.1002/imt2.70111)
Supplement: Supplementary file 1 [file IMT2-5-e70111-s002.docx]

**Supporting information to**

***Curcuminoids Amplify Host Innate Antiviral Immunity via the CRYAB-RBM26 Axis in Viral Infection***

Running head: Curcuminoids modulate the CRYAB–RBM26 axis

Tingting Chen^1,2,3†^, Xiang Wang^1,4†*^, Guangyuan Zhang^1,3†^, Lu Wang^3^, Junhao Wan^1^, Lirong Tan^5^, Xinyue Fan^3^, Siqin Meng^3^, Zhixing Jin^3^, Yuzhang Liang^3^, Haoze Li^3^, Ziyuan Wan^3^, Xiaotong Xu^3^, Yan Zhang^3^, Li Qin^2^, Anna Malashicheva^6^, Yu Du^1*^, Guangchao Zang^1,2,3*^ and Guixue Wang^2,7,8*^

1 Department of Orthopedics, The Second Affiliated Hospital of Chongqing Medical University, Chongqing, 400010, China

2 Institute of Panvascular Biology, JinFeng Laboratory, Chongqing, 401329, China

3 Pathogen Biology and Immunology Laboratory, Lab Teaching & Management Center, Chongqing Medical University, Chongqing, 400016, China

4 National Key Laboratory for Development and Utilization of Forest Food Resources, Institute of Chemical Industry of Forest Products, Chinese Academy of Forestry; Nanjing 210042, China

5 Institute of Medical College, Guizhou University, Guiyang, 550025, China

6 Institute of Cytology, Russian Academy of Science, Tikhoretskiy Prospect 4, 194064 St. Petersburg, Russia

7 Key Laboratory of Biorheological and Technology of Ministry of Education, National Local Joint Engineering Laboratory for Vascular Implants, Bioengineering College of Chongqing University, Chongqing, 400044, China

8 School of Chinese Medicine, Hubei University of Chinese Medicine, Wuhan, 430065, China

† Tingting Chen, Xiang Wang and Guangyuan Zhang contributed equally to this work and share the first authorship.

* Correspondence: Xiang Wang, wangxiang19930424@caf.ac.cn; Yu Du, duyu@hospital.cqmu.edu.com; Guangchao Zang, zangguangchao@cqmu.edu.cn; Guixue Wang, wanggx@cqu.edu.cn

**Supplementary Figures**

**Figure S1. Antiviral efficacy of CUR and DMC in the EV-D68 mouse model. (A)** Representative histological sections of lung, brain, liver and spleen tissues. Inflammatory infiltrates are indicated by black arrows. Scale bars: 50 μm (overview), 10 μm (magnified view). Normal mice without treatment were used as the MOCK group. **(B)** The relative foldchange (FC) of viral *2A* gene level. Relative mRNA levels of the viral *2A* gene in brain tissues at 14 days post-infection (dpi), as quantified by RT‒qPCR. The results are expressed as the mean ± SD. Statistical significance: ***p*  < 0.05, ****p*  < 0.001, ns, not significant.

**Figure S2. Upstream regulators of CRYAB modulated by CUR/DMC. (A)** Cycloheximide (Chx) chase assay to detect the half-life of the CRYAB protein. RD cells in the logarithmic growth phase (70–80% confluency) were transfected with the CRYAB plasmid. At 24 h post-transfection, the medium was replaced with fresh medium containing CUR (40 μM), DMC (20 μM), or DMSO (negative control). The cells in the positive control group were treated with MG132 (10 μM). After 24 h of treatment, all the cells were incubated with Chx, and total cellular proteins were extracted at 0, 2, 4, 6, and 8 hours post-Chx treatment for WB analysis, *n* = 3; the graphs show the mean ± SD. ns, *p* > 0.05; ***p* < 0.01. **(B)** Regulation of CRYAB transcriptional levels by CUR/DMC. RD cells in the logarithmic growth phase (80–90% confluency) were cultured in fresh medium supplemented with CUR (40 μM), DMC (20 μM), CUR (40 μM) plus the HSF inhibitor DTHIB (5 μM), DMC (20 μM) plus the HSF inhibitor DTHIB (5 μM), or DMSO (negative control). After 24 h of treatment, total cellular RNA was extracted, and CRYAB transcriptional levels were detected via RT‒qPCR, with GAPDH serving as the internal control, *n*=3; the graphs show the mean ± SD. ns, *p* > 0.05; ***p* < 0.01, ****p* < 0.001. **(C)** Molecular docking analysis of HSF with CUR and DMC. **(D)** Relative mRNA levels of CRYAB in RD cells transfected with siCRYAB or scrambled control RNA (500 pmol RNA/5 μL transfection reagent) for 48 h. Total RNA was extracted and analysed via RT‒qPCR, *n* = 3; the graphs show the mean ± SD. ns, *p* > 0.05; *****p* < 0.001. **(E)** Quantification of viral protein expression in the CRYAB-overexpressing and vector control groups via proteomic analysis. The graphs show the mean ± SD. ****p* < 0.001.

**Figure S3. Differential gene expression and molecular docking analysis in the HepG2-HBV infection model. (A)** Venn diagram showing differentially expressed genes regulated by CUR and DMC. Shared genes were designated as the CUR_DMC_hub module, while uniquely regulated genes were defined as CUR-specific or DMC-specific. **(B)** Reactome pathway enrichment analysis of genes co-regulated by CUR and DMC, as well as their respective specific gene sets. **(c)** Molecular docking analysis of CRYAB with CUR and DMC. The human CRYAB protein structure (PDB ID: 3L1G) was obtained from the PDB database (https://www.rcsb.org/). Protein preprocessing was performed using PyMOL, including removal of water molecules, hydrogen addition, charge assignment, and native ligand deletion. Global docking was performed using AutoDock Vina 1.2.7. The docking box was centered at x = 153.023, y = 77.906, z = 113.731, with dimensions of 47.25 Å × 47.25 Å × 47.25 Å and grid spacing set to 0.375 Å. **(d)** Relative mRNA levels of CRYAB in RD cells transfected with siCRYAB or scrambled control RNA (500 pmol RNA / 5 μL transfection reagent) for 48 h. Total RNA was extracted and analyzed via RT-qPCR, *n*=3; graphs show mean ± SD. ns, *p* > 0.05; *****p* < 0.001. **(e)** Quantification of viral protein expression in CRYAB-overexpressing and vector control groups based on proteomic analysis. Graphs show mean ± SD. ***p* < 0.05; ****p* < 0.001.

**Figure S4. CRYAB subcellular localization and interaction with importin β during EV-D68 infection. (A)** Volcano plot of differentially abundant metabolites between CRYAB-overexpressing and vector control cells. Immunofluorescence staining of CRYAB protein in RD cells. Cells were either mock-treated, infected with EV-D68, or infected with EV-D68 following pretreatment with importazole (40 μM) for 1 h. CRYAB is shown in green, and nuclei are stained with DAPI (blue). Scale bar = 30 μm **. (B)** Co-immunoprecipitaion assay. RD cells were transfected with CRYAB.HA. Cell lysates were immunoprecipitated with anti-HA antibody or control IgG, followed by western blot analysis for importin β, HA, and GAPDH (loading control).

**Figure S5. Mult-omics analysis of the downstream signal pathway targeted by CRYAB. (A)** Volcano plot of differentially abundant metabolites between CRYAB-overexpressing and vector control cells. **(B)** KEGG pathway analysis of metabolites upregulated or downregulated by CRYAB overexpression. **(C)** KEGG enrichment map. Enrichment analysis of DEGs was performed via the hypergeometric test, with *P* values adjusted via the Benjamini‒Hochberg (BH) method. Terms with adjusted values (*P*adjust < 0.05) were considered significantly enriched. The top 10 genes ranked by log_2_FC per term/pathway are shown. **(D)** EggNOG functional classification of CRYAB-regulated differentially expressed proteins (DEPs). DEPs were analysed via EggNOG (http://eggnog5.embl.de//app/home). Percentage = (number of pathway-associated proteins/total DEPs) × 100%.

**Figure S6. Molecular docking analysis of CRYAB-interacting proteins and PTM-related protein alterations. (A)** Mapping of DEPs to metabolic pathways via iPath 3.0. Red indicates proteins upregulated by CRYAB, green indicates downregulated proteins, and blue indicates proteins directly interacting with CRYAB. **(B)** Weighted docking scores (lowest binding energy) of CRYAB with hub-up-IP, hub-down-IP, and STRING-derived reference proteins. LigPlot+ (v2.2) was used for visualization. Hydrogen bonds are highlighted in yellow; hydrophobic interactions are highlighted in green. The black labels denote UniProt IDs from the STRING database; red and green indicate interaction proteins whose expression is up- or downregulated by CRYAB, respectively. **(C)** Heatmap of differential posttranslational modifications (phosphorylation and ubiquitination) in the CRYAB-overexpressing group vs. the vector control group. Proteins were selected on the basis of *p* < 0.05 and |log_2_FC| > 1.2. V-68 denotes EV-D68 infection after vector transfection; C-68 denotes EV-D68 infection after CRYAB overexpression. **(D)** Pathway enrichment analysis of the hub genes identified through integrated proteomic and PTM datasets. **(E)** Two-dimensional interaction map of CRYAB with RBM26. Green: RBM26 residues; blue: CRYAB residues; lines represent hydrogen bonds; arcs represent hydrophobic interactions.

**Figure S7. Identification of the interaction between CRYAB and RBM26. (A)** Functional conservation analysis of the interaction sites between CRYAB and RBM26. Red stars indicate highly conserved interaction sites. **(B)** Construction of CRYAB point mutation plasmids on the basis of the identified interaction sites. A Co-IP assay was performed to verify the interaction between the CRYAB mutant plasmid series and RBM26. RD cells in the logarithmic growth phase (70–80% confluency) were cotransfected with the RBM26 plasmid and each CRYAB mutant plasmid. At 48 h post-transfection, the cells were infected with EV-D68 (MOI = 1). Total cellular proteins were extracted at 24 h post-infection for the Co-IP assay, in which the HA antibody was used for IP. The protein levels of HA, Flag, and GAPDH were detected by WB. (**C)** Regulation of RBM26 ubiquitination by CRYAB mutant plasmids. After the RBM26 plasmid was cotransfected with each CRYAB mutant plasmid, IP was performed via an anti-Flag antibody, and the ubiquitination level of RBM26 was detected via an anti-ubiquitin (Ub) antibody. **(D)** Identification of RBM26 ubiquitination sites. The ubiquitination sites of RBM26 and their conservation were analysed via quantitative proteomics (protein modification omics), and corresponding point mutation plasmids were constructed. RBM26-associated proteins were pulled down with a Flag antibody, followed by WB detection with antibodies against Ub, Flag, and GAPDH. **(E)** Identification of the type of EV-D68-mediated ubiquitin chain on RBM26. RD cells in the logarithmic growth phase were cotransfected with the RBM26-Flag plasmid and either the wild-type (WT) Ub plasmid or the K48-linked Ub mutant plasmid. At 48 hours post-transfection, the cells were infected with EV-D68 (MOI = 1). Total cellular proteins were extracted at 24 hours post-infection, and the level of ubiquitinated RBM26 was detected via an IP‒WB assay.

**Figure S8. Investigating the contribution of CRYAB to RBM26. (A)** The relative mRNA level of viral *2A* gene. RD cells were transfected with either RBM.Flag plasmid or empty vector control. At 48 h post transfection, cells were infected with EV-D68 (MOI = 1). Total RNA were extracted at 24 h post-infection and subjected to RT-qPCR analysis for the viral *2A* gene. Gene expression was normalized to the internal control GAPDH. Data are presented as mean ± SD; *n* = 3, ns, not significant (*p* > 0.05); **0.001 < *p* < 0.05; ****p* < 0.001. **(B)** RBM26 mRNA expression. RD cells were cultured in 10 cm dishes to 50–60% confluency (1×10⁶ cells) in antibiotic-free medium. Transfection was performed using DEPC-treated glucose-containing water for siRNA dilution (300 pmol siRNA/15 µL transfection reagent). After 48 hours of siRNA transfection, cells were further transfected to overexpress RBM26 or RBM26+CRYAB (12 µg/dish). siRNA-negative control with empty vector, SiN siRBM26 with empty vector, SiR, siRBM26 with RBM26 overexpression, siR-R, siRBM26 with RBM26 and CRYAB co-overexpression, SiR-C. After 48 hours of overexpression, cells were infected with virus, and total RNA was collected 24 hours post-infection for transcriptome sequencing. Data are presented as mean ± SD; *n* = 3, ns, not significant (*p* > 0.05); ***p* < 0.05; ****p* < 0.001. **(C)** Reactome pathway enrichment of DEGs. Differentially expressed genes (DEGs) were identified in SiRBM26 (SiR) vs. siRNA negative control (SN), SiRBM26 + RBM26 (R) vs. SN, SiRBM26 + RBM26 + CRYAB (RC) vs. SN. Pathways with adjusted *p*-value <0.05 were considered significantly enriched. **(D)** GSVA results for RBM26 group. Gene Set Variation Analysis (GSVA) was performed to transform gene-level expression matrices into pathway-level expression profiles. The horizontal axis indicates the t-value (magnitude of expression difference between samples), and the vertical axis lists pathway names. Significantly enriched gene sets were defined as *P*adjust < 0.05 and |t| ≥1.96. Blue represents significantly upregulated pathways; green indicates significantly downregulated ones. **(E)** GSVA enrichment in the RBM26+CRYAB group. **(F)** Venn analysis of overlapping genes. Venn analysis was conducted to compare up-and downregulated genes in the SiR group with those reversed upon RBM26 re-expression.

**Figure S9. WGCNA-based identification of trait-associated gene modules. (A)** Module detection and correlation with traits. Left: Gene dendrogram and module color assignment by WGCNA. Right: Correlations between gene modules and experimental traits; correlations with *p* < 0.05 were considered significant. **(B)** Metabolic pathway analysis. iPath3.0 (http://pathways.embl.de) was used to visualize metabolic pathways. Nodes represent metabolites; edges denote enzymatic reactions. Pink, pathways annotated from the WGCNA_SiR gene set; light green, pathways annotated from the WGCNA_r_rc gene set; blue, shared pathways annotated in both gene sets. **(C)** Log_2_FC values of key targets at the transcript and protein levels. Four overlapping genes were identified from the RBM26_CRYAB_unique AS-related gene set, which included both transcriptomic and proteomic DEGs associated with CRYAB overexpression. The expression levels were visualized via Origin software.

**Figure S10. Identification of downstream targets of RBM26 and its regulatory mechanisms in the IFN-I pathway. (A)** Alternative splicing of CMPK2. Visualization of alternative splicing (AS) events in *CMPK2* between the SiR and SiR+RBM26 groups. The upper panel displays the read coverage depth across samples (Y-axis) mapped to the genomic coordinates of *CMPK2* (X-axis), with colored lines indicating splice junctions supported by read counts. Sample names are shown on the left, with different colors representing group assignments. The lower panel presents transcript IDs and schematic representations of AS events in each isoform. **(B)** Expression levels of *CMPK2* transcripts. The FPKM values of individual *CMPK2* transcripts were compared across groups after normalization to those of the NC group. Transcript expression differences were assessed by letter-based grouping: identical letters indicate no significant difference, whereas different letters denote statistical significance. **(C)** Total *CMPK2* expression. Overall *CMPK2* gene expression (*n* = 3) was quantified as FPKM values and was presented as the mean ± SD. Statistical analysis: **0.001 < *p* < 0.05; ****p* < 0.001; ns, not significant. **(D)** Quantitative PCR analysis of RBM26 (left) and CMPK2 (right) mRNA levels. RD cells were treated with DMSO, CUR, DMC for 48 h, Total RNA was then extracted, and the relative mRNA levels of *RBM26* and *CMPK2* were determined by RT-qPCR. The data were presented as the mean ± SD (*n* = 3). Statistical significance was determined by one-way ANOVA: **p* < 0.05; ***p* < 0.01; ****p* < 0.001. **(E)** Validation of *CMPK2* knockdown efficiency in RD cells using three distinct siRNA sequences. RD cells were transfected with siRNA (SiNC), or one of three independent siRNAs targeting *CMPK2* (siCMPK2-1,-2,-3). At 48 h post-transfection, total RNA was extracted. The relative mRNA level of CMPK2 was analyzed by RT-qPCR, normalized to *GAPDH*, and is presented as the fold change (FC) relative to the siNC group. The data were presented as the mean ± SD (*n* = 3). Statistical significance was determined by one-way ANOVA: **p* < 0.05; ***p* < 0.01; ****p* < 0.001. **(F)** The relative mRNA level of viral *2A* gene. RD cells were transfected with siRNA (SiNC), or siCMPK2, siRBM26. At 48 h post-transfection, those transfected cells were infected with EV-D68 (MOI = 1). At 24 h post-infection, total RNA was extracted. The relative mRNA level of *2A* gene was analyzed by RT-qPCR, normalized to GAPDH, and is presented as the fold change (FC) relative to the siNC group. The data were presented as the mean ± SD (*n* = 3). Statistical significance was determined by one-way ANOVA: **p* < 0.05; ***p* < 0.01; ****p* < 0.001. **(G)** Liquid chromatography analysis of cytidine and uridine and corresponding standard curves. Upper panels represented the separation and detection of cytidine (left) and uridine (right). External standard curve used for absolute quantification, plotting the known concentration of each standard against the integrated chromatography peak area. **(H)** Quantification of uridine levels in cell lysates under different treatments. The concentration of uridine was determined by LC-MS based on the standard curve in (G) and normalized to total protein content. The data were presented as the mean ± SD (*n*=3). Statistical significance was determined by one-way ANOVA: **p* < 0.05; ***p* < 0.01; ****p* < 0.001. **(I)** Cell viability assay. RD cells were seeded in a 96-well plate and allowed to adhere. Cytidine or uridine was then added to reach final concentrations of 1, 2.5, 5, 10, 20, or 40 μM. After 36 h, cell viability was assessed via the CCK-8 assay. **(J)** Standard curve for absolute quantification of mtDNA copy number. A ten-fold serial dilution of ND1 plasmid was used as a template to generate a standard curve by qPCR. The cycle threshold (Ct) values are plotted against the logarithm of the known template copy number. The liner regression equation (y = -0.3489x + 14.475) and the coefficient of determination (R^2^ = 0.9906) are shown.

**Figure S11. RBM26 deficiency alters nucleotide metabolism and antiviral signaling, impairing the metabolic support for EV-D68 replication. (A)** Relative mitochondrial DNA (mtDNA) levels. RD cells were seeded into 6-well plates and treated as follows: i. Cells were infected with EV-D68 (MOI = 1), followed by medium replacement after 2 h with fresh culture medium containing DMSO, 40 μM CUR, or 20 μM DMC. After 12 h of infection, total DNA was extracted, and mtDNA/nuclear DNA levels were measured via qPCR. ii. Cells transfected with SiR for 24 h were subsequently transfected with pRBM26-Flag or empty vector. After 48 h, the cells were infected with EV-D68 for 12 h prior to DNA extraction and qPCR analysis. The MOCK group served as an uninfected control; DMSO was used as the control for drug-treated infected cells. **(B)** RBM26 partially rescues the reduction in mtDNA copy number caused by CRYAB knockdown. RD cells were transfected with SiNC, SiCRYAB, or SiCRYAB in combination with an RB26 overexpression plasmid. The relative mtDNA copy number was determined by qPCR using the standard curve from (S11J) and normalized to nuclear DNA. Data are presented as FC relative to SiNC group, The data were presented as the mean ± SD (*n* = 3). Statistical significance was determined by one-way ANOVA: **p* < 0.05; ***p* < 0.01; ****p* < 0.001. **(C)** cGAMP levels. RD cells were seeded in a 6-well plate. The MOCK group served as the normal control. The experimental groups were divided into six conditions. For the three groups, poly(dA:dT) (1 µg/well) was added to the cells, and after 2 h, the medium was replaced with fresh culture medium containing DMSO, 40 μM CUR, or 20 μM DMC. In the remaining three groups, the cells were infected with EV-D68 (MOI=1), after which the medium was replaced with fresh culture medium containing DMSO, 40 μM CUR, or 20 μM DMC. The supernatants from the cell lysates were collected, and the cGAMP levels were measured via ELISA. The data are presented as the mean ± SD (*n* = 3). Statistical significance was assessed by one-way ANOVA: **p* < 0.05; ***p* < 0.01. **(D)** Western blot analysis of STING-TBK1-IRF3 signalling activation. RD cells were seeded in 6-well plates and subjected to drug treatments or plasmid transfection. Poly(dA:dT) (1 µg/well) was used as a positive control. The experimental groups included uninfected, EV-D68-infected, CUR-treated, and DMC-treated cells. The data are shown as the mean ± SD (*n* = 3). Statistical analysis was performed via one-way ANOVA: **p* < 0.05; ***p* < 0.01; ****p* < 0.001; *****p* < 0.0001. **(E)** Diagram of the induction and effector phases of type I interferons. **(F)** ELISA quantification of IFN-α and IFN-β secretion. RD cells were seeded in 6-well plates and transfected with empty vector or pRBM26 or cotransfected with pRBM26 and CRYAB (6 μg/well). At 48 h posttransfection, the cells were infected with EV-D68 (MOI = 1). The supernatants were collected at 18 h post infection for ELISA. Uninfected RD cells treated with poly(I:C) (30 nM/well) for 12 h served as the positive control. The data represent the mean ± SD (*n* = 6). Statistical significance was assessed via one-way ANOVA: **p* < 0.05; ***p* < 0.01; *****p* < 0.0001. **(G)** Western blot analysis of STAT1. RD cells were seeded into 6-well plates and divided into six groups, with three replicates in each group. After cell adhesion, the following transfection treatments were performed: the DMSO group, CUR group, and DMC group were transfected with an empty vector; the RBM26 group was transfected with pRBM26. Flag plasmid (3 μg); the CRYAB group was transfected with pCRYAB. HA plasmid (3 μg). At 36 h posttransfection, the cells were cultured in serum-free, antibiotic-free medium and infected with the EV-D68 virus (MOI = 1). Two hours after viral infection, the medium was replaced with normal culture medium. For the DMSO, RBM26, and CRYAB groups, DMSO was added to the medium, whereas the CUR and DMC groups received 40 μM and 20 μM drugs, respectively. After being cultured for 12 h, the cells were collected for total protein extraction. The data are shown as the mean ± SD (*n* = 3). Statistical analysis was performed via one-way ANOVA: ***p* < 0.01. **(H)** Relative ISG mRNA levels. RD cells were seeded into 6-well plates at 70–80% confluency and divided into 8 groups. The cells were transfected with the shCRYAB plasmid (SiC), while the control group was transfected with the shNC vector (siNC). After 24 h, the medium was replaced with complete culture medium. The SiNC and SiC groups were transfected with an empty vector, whereas the SiC-RBM26 group was transfected with pRBM26. Flag plasmid (3 μg), and the SiC-CRYAB group with pCRYAB. HA plasmid (3 μg). After 24 h, the medium of the cells was changed to serum- and antibiotic-free medium, and the cells were subsequently infected with EV-D68 (MOI = 1) for 2 h. Following infection, one set of the SiNC, SiC, SiC-RBM26, and SiC-CRYAB groups was treated with complete medium containing 20 μM DMC, and the other set was treated with 40 μM CUR. After culture for 12 h, the cells were collected, and total mRNA was extracted for RT‒qPCR detection. The data were presented as the mean ± SD (*n* = 3). Statistical significance was determined by one-way ANOVA: ****p* < 0.001. **(H)** The relative expression levels of *CRYAB* and three classic ISGs, *IFIT1*, *OAS1*, and *MX1* in the brain tissue were measured by RT-qPCR. The data were presented as the mean ± SD (*n* = 3). Statistical significance was determined by one-way ANOVA: **p* < 0.05; ***p* < 0.01; ****p* < 0.001.
